# Development and Application of Liquid Crystals as Stimuli-Responsive Sensors

**DOI:** 10.3390/molecules27041453

**Published:** 2022-02-21

**Authors:** Sulayman A. Oladepo

**Affiliations:** 1 Department of Chemistry, King Fahd University of Petroleum and Minerals, Dhahran 31261, Saudi Arabia; saoladepo@kfupm.edu.sa; Tel.: +966-13-860-7103; Fax: +966-13-860-4277; 2 Interdisciplinary Research Center for Advanced Materials (IRC-AM), King Fahd University of Petroleum and Minerals, Dhahran 31261, Saudi Arabia

**Keywords:** liquid crystals, thermotropic liquid crystals, LC-based sensors, 5CB liquid crystals, homeotropic alignment, planar alignment, LC/aqueous interface, polarized optical (POM) image

## Abstract

This focused review presents various approaches or formats in which liquid crystals (LCs) have been used as stimuli-responsive sensors. In these sensors, the LC molecules adopt some well-defined arrangement based on the sensor composition and the chemistry of the system. The sensor usually consists of a molecule or functionality in the system that engages in some form of specific interaction with the analyte of interest. The presence of analyte brings about the specific interaction, which then triggers an orientational transition of the LC molecules, which is optically discernible via a polarized optical image that shows up as dark or bright, depending on the orientation of the LC molecules in the system (usually a homeotropic or planar arrangement). The various applications of LCs as biosensors for glucose, protein and peptide detection, biomarkers, drug molecules and metabolites are extensively reviewed. The review also presents applications of LC-based sensors in the detection of heavy metals, anionic species, gases, volatile organic compounds (VOCs), toxic substances and in pH monitoring. Additionally discussed are the various ways in which LCs have been used in the field of material science. Specific attention has been given to the sensing mechanism of each sensor and it is important to note that in all cases, LC-based sensing involves some form of orientational transition of the LC molecules in the presence of a given analyte. Finally, the review concludes by giving future perspectives on LC-based sensors.

## 1. Introduction

Liquid crystals are a class of chemical substances that exist in intermediate states between crystalline solids and liquids [1,2,3,4,5]. They thus share the anisotropic properties of crystalline solids as well as fluid properties of isotropic liquids [5,6,7]. They exhibit various phases due to the noncovalent and orientation-dependent interactions that exist between their molecules [1,2,8,9]. Their anisotropic properties and molecular orientations are responsible for their delicate sensitivity to external stimuli, including light, temperature, mechanical shear, electric field, magnetic field and surface interactions with foreign molecules [10,11,12,13,14,15,16]. Such delicate and exquisite sensitivity is responsible for their exploitation as stimuli-responsive materials in various applications such as display and visualization technology, photovoltaics, optoelectronics, sensors and material science [17,18,19,20,21]. As a consequence, intense research efforts are directed at using LCs as sensitive, fast-response and low-cost sensor materials [7,21,22].

The LCs used for sensing applications are multivarious and extensive because of their self-assembly characteristics and functional diversity [22,23]. The LCs used for various sensing applications reviewed in this paper are for the most part 4-cyano-4′-pentylbiphenyl (5CB) [24,25], which is a member of the alkylcyanobiphenyl class of LCs, though a few applications make use of other kinds of LCs. The delicate nature of the force balance that exists in LCs organization forms the basis of all sensing schemes. Therefore, given their stimuli-responsive nature, LCs can be regarded as materials that respond rapidly to the presence of foreign species or the occurrence of binding events in their vicinity [21]. LCs also have the ability to quickly amplify and transform molecular events occurring in their vicinity, however minute, into macroscopic measurable signals. Thus, the basic properties of LCs exploited in all sensing applications are the delicate sensitivity to all kinds of external stimuli that manifests mostly in form of optical visualization and the high speed with which such responses are presented. In addition, most of the sensing formats so far reported are easily fabricated. These factors represent significant advantages for which LCs have been harnessed for various applications. In comparison with conventional sensing technologies that require extensive manufacturing and high-precision instruments, LC-based sensors are superior due to their sensitivity, easy fabrication, rapid response and low costs. Hence, they have been extensively employed as signal reporters and amplifiers in various sensing applications [22]. 

**Scheme 1:**
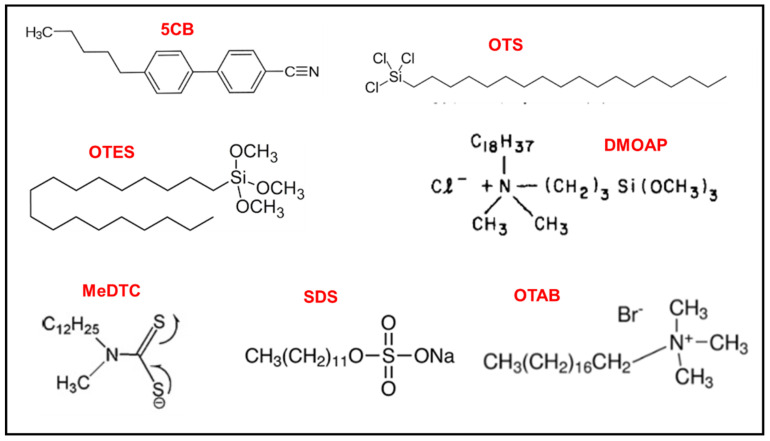
Chemical structures of selected compounds.

In general, three major formats are used in the fabrication of LC-based sensors. One format involves applying the LC molecules on specially treated glass substrates while suspending and confining them in transmission electron microscopy (TEM) grids, usually doped with LC/air interface assembly molecules and the whole unit is then immersed in a solution of the analyte. In some cases, an independent analyte recognition/interaction molecule may be added to the mix if the presence of the analyte is unable to perturb the LC alignment in a significant way with the original chemical composition. In many cases, the surfactants originally added to the sensor medium have the right chemistry that interacts with the analyte and triggers realignment of the LC molecules. Such analyte recognition/interaction molecule will trigger reorientation of the LC molecules in the presence of the analyte. However, if the analyte is absent, such reorientation will not take place. Treatment of the glass substrate is meant to provide primary ordering for the LCs. Chemicals such as octadecytrichlorosilane (OTS), *N*,*N*-dimethyl-*N*-octadecyl-3-aminopropyl trimethoxysilylchloride (DMOAP), or octadecyltrietoxysilane (OTES) may be used for this purpose [21,22,26,27]. LC/air interface assembly molecules are also added to the mix. These are usually surfactants or phospholipids, which are amphiphilic molecules with polar head groups and aliphatic alkyl chains [21,27]. Such molecules fall in the category of phospholipids, ionic and non-ionic surfactants and LC polymers. Examples include N-methyl-N-dodecyldithiocarbamate (MeDTC), sodium dodecyl sulfate (SDS), Tween-20, alkyl trimethylammonium bromide (CnTAB), octadecyltrimethyl ammonium bromide (OTAB), (hydroxyundecyl)trimethyl ammonium bromide (HTAB), tetra(ethylene glycol) monotetradecyl ether (C14E4), tetra(ethylene glycol) monododecyl ether (C12E4) and amphiphilic block copolymers such as poly(acrylicacid-b-4-cynobiphenyl-4-oxyundecylacrylate), i.e., PAA-b-LCP [21,27,28,29,30,31]. A previous review has presented the use of a variety of polyelectrolytes to functionalize the LC-aqueous interface, yielding an interesting and promising class of interfaces for applications in chemical and biological sensing [32]. The chemical structures of 5CB and selected chemicals used for treating glass substrates as well as LC/air interface assembly molecules are shown in Scheme 1 below.

**Figure 1:**
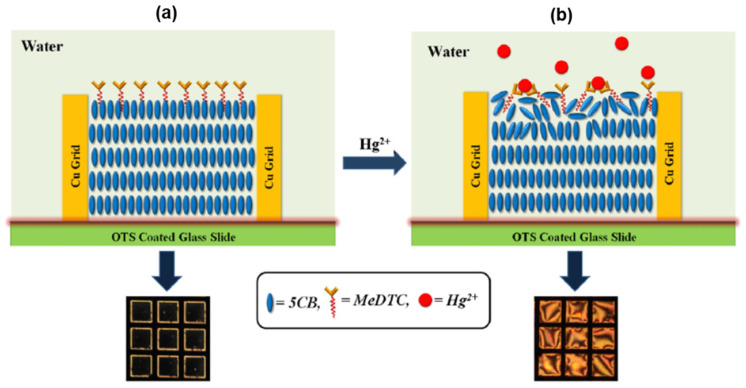
Alignments of LC molecules: (**a**) homeotropic alignment of LC molecules that gives rise to a dark image, and (**b**) disordered/planar alignment that allows some polarized light to go through, giving rise to a bright image. Reprinted with permission from ref. [27]. 2015 Elsevier.

The second format is a sandwich format that simply involves another glass slide that is placed on top of the TEM grid assembly of the first format, such that the LC and analyte recognition/interaction molecules are sandwiched between two glass plates before being immersed in the analyte solution [21,22,26]. In both first and second formats, the LC molecules can adopt a homeotropic alignment (vertical orientation) with respect to the glass substrate or they may adopt a planar orientation, depending on the environment or chemical composition surrounding the LC molecules. LCs have a birefringence property, which could be observed with a polarized optical microscope [5,33]. Thus, the setup is observed under cross polarizers in an optical microscope, where the homeotropic alignment of the LCs appears dark and planar orientation appears bright. Dark or bright field in the optical response is dictated by the chemical composition or interaction at the LC/aqueous interface. Usually, the sensing mechanism is an optical transition from a dark to a bright image or vice versa when the sensor is immersed in the analyte solution and viewed under cross polarizers with an optical microscope. As depicted in Figure 1, the sensor assembly of 5CB molecules doped with MeDTC was applied to copper grids. The MeDTC was added to the LC because it can self-assemble at the LC/aqueous interface and induce a homeotropic anchoring of 5CB. This homeotropic alignment of the LC molecules gives a dark image as shown. However, when immersed in a solution of Hg(II) ions, the ions interact with the dithio-chelating group in the MeDTC and this interaction disrupts the homeotropic alignment of the 5CB liquid crystal at the LC-water interface, resulting in a bright image.

**Figure 2:**
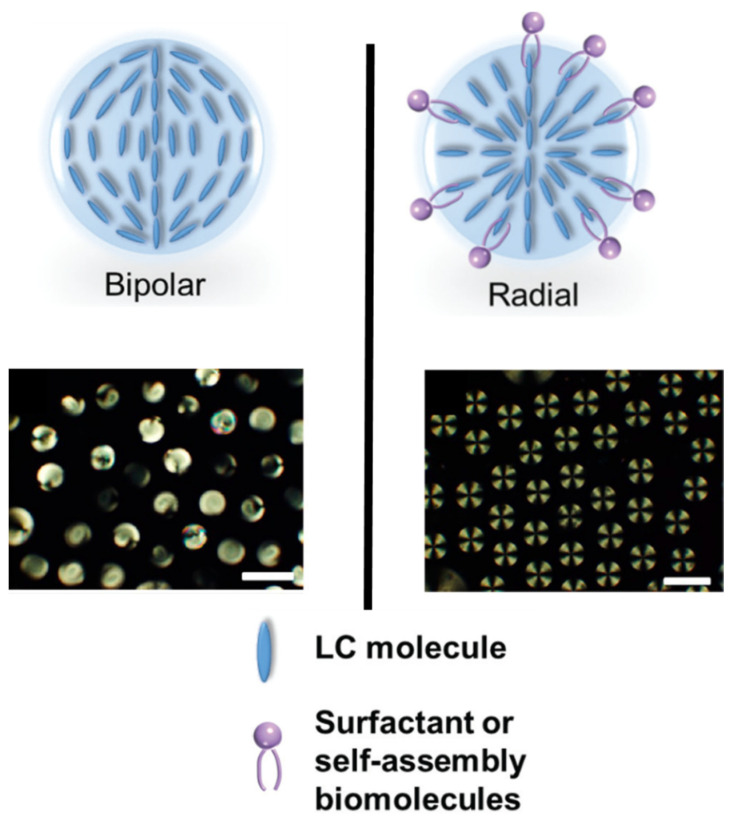
Bipolar alignment of LC molecules (**left** panel) and radial orientation of LC molecules (**right** panel). The corresponding polarized optical microscopy (POM) image is shown below each. Typical diameter of a LC microdroplet is about 10–100 µm. Reprinted with permission from refs. [22,36]. 2021 The Royal Society of Chemistry and 2013 American Chemical Society.

The third format involves aqueous droplet interfaces where microdroplets of LCs are suspended in bulk water [22,34]. Biomolecules can cause the reorientation of LCs in the microdroplet due to the absorption from surfaces of LC droplets [22]. In this format, the size of the droplet is an important determinant of sensitivity since the large surface-area-to-volume ratio of LC microdroplets enables full reaction at the LC/aqueous interface thereby producing a lower detection limit [35]. Furthermore, the configuration of the LCs depends on the diameter of the droplet, typically around 10–100 µm [36,37,38]. Ordering of LC molecules can produce either a radial or bipolar configuration, again depending on the chemical composition of the environment. In a manner similar to the dark versus bright images obtainable in the first two formats, this format presents either radial or bipolar image and the optical response can be from one configuration to the other (Figure 2). Any one of the three formats is usually employed for LC-based sensing systems. It should be mentioned that almost all (if not all) the LC-based sensors make use of polarized optical microscopy (POM) image as the detection method.

The crystalline properties of LCs involve long-range orientational order, and such orientational ordering of LC molecules gives rise to anisotropic properties [38]. The simplest LC phase is the nematic phase in which the molecules possess orientational order of their long molecular axis but no positional order of their centre of mass. This preferred orientation, which represents the average orientations of all the LC molecules in the nematic phase is described by a director, *n* [7,38]. The orientation of the LC molecules is sensitive to external stimuli such as electric or magnetic field, shear, stress and chemical analytes, which can disrupt the preferred orientation, leading to a distortion of the director field. Such interactions between the external stimuli and LC molecules produce an elastic restoring force and thereby increase the free energy of the system [7,22]. The free energy (elastic energy) of the LC in the nematic phase can be described by the Frank–Oseen equation:

(1) $$F_{e}=\frac{1}{2}\left[K_{11}{\left(\nabla \cdot n\right)}^{2}+K_{22}{\left(n\cdot \nabla \times n\right)}^{2}+K_{33}{\left(n\times \nabla \times n\right)}^{2}\right]$$

where *F_e_* is the elastic energy, *K*_11_, *K*_22_ and *K*_33_ are the Frank’s elastic constants associated with splay, twist and bend, respectively. This elastic free energy *F_e_* is basically the energetic penalty associated with deviations of the director from its preferred alignment in the nematic phase [38]. The extent of disruption of the preferred orientation of the LC molecules by an external stimulus (e.g., a chemical analyte) will determine how much elastic restoring force will be generated from the LC. So, the elastic free energy dictates the LC orientations, and therefore orientational transitions in LC form the basis for sensing.

In addition to long-range orientational order, LC also exhibits anisotropy. Birefringence is arguably the most significant and well-known anisotropic property [7]. Birefringence refers to the dependence of the refractive index on the direction of light propagation. Therefore, due to their birefringence, LCs split plane polarized light into two orthogonal components of ordinary and extraordinary rays upon entering a nematic LC medium. The two component rays will experience different velocities in the LC medium and therefore different refractive indices: the ordinary refractive index, *n_o_*, and the extraordinary refractive index, *n_e_* [7,22,38,39]. The *n_o_* and *n_e_* correspond to a light beam polarization perpendicular and parallel to the optical axis, respectively. This phenomenon leads to a phase difference when the light beam emerges from the LC medium. This phase difference is known as optical retardation, *δ*, and it is given by [22,39,40,41,42]:

(2) $$\delta =\frac{2\pi d}{\lambda }\left(n_{e}-n_{o}\right)$$

where *λ* is the wavelength of light, *d* is the thickness of the medium, and (*n_e_* − *n_o_*) is the birefringence (Δ*n*). Therefore, LC systems are able to manipulate light polarization due to birefringence. Optical retardation is the reason why the polarization state changes upon passing through an anisotropic medium. The interference between the two orthogonal component rays is responsible for the colourful optical textures of LC that are observed via cross polarizers. The director configuration of LCs confined within certain geometries can be determined by using polarized optical microscopy by looking at the patterns generated through the interaction of light with the LC. Therefore, when LC molecules are sandwiched between two cross polarizers, the intensity of the transmitted light, *I*, is given by [7,22,40,43,44]:

(3) $$I=I_{o}sin^{2}2\varphi sin^{2}\frac{\delta }{2}$$

where *I_o_* is the light intensity exiting the first polarizer, and *φ* is the sample position with respect to the polarizer. This equation shows that for the homeotropic alignment of the LC, the transmitted light, *I* = 0 (because *δ* = 0) and so the optical texture appears dark under cross polarizers, whereas the optical texture appears bright under cross polarizers for the planar arrangement of the LC because *I* ≠ 0 (for *δ* ≠ 0). 

The analyte or target substance for which LC-based sensors have been developed are numerous. This current review presents most of these types of applications. Several reports have appeared in the literature where LCs have been used as biosensors, which are devices that can provide selective quantitative or semi-quantitative analytical information using a biological recognition element [45]. These sensors are meant to detect substances that are either essential or inimical to life [34]. Several LC-based sensing platforms have been reported for biologically relevant substances such as glucose or sugars [36,37,46,47,48,49,50], amino acids and proteins [50,51,52,53,54,55,56], urea [35,57,58], enzymes [59,60,61,62], DNA [63,64], antigens [65,66], disease biomarkers [67,68], cells and viruses [69,70]. Similarly, there is extensive literature on the application of LCs as sensors for heavy metals [71,72,73], toxic agents [15,74,75,76] and gases [77,78,79]. In the same vein, there are LC-based sensors developed for pH monitoring [80,81], as strain sensors [82] and as a radiation dosimeter [83], while LC is also being used as a temperature sensor [84]. This review discusses all these areas of application, with a particular emphasis on the sensing mechanism in each case. Future perspectives and direction in the field of LC-based sensing are also presented at the end.

## 2. LC-Based Biosensors

One of the most common applications of LCs is as a platform for sensing biomolecules such as ions, sugars, amino acids, proteins, nucleic acids, antigens, antibodies, dissolved gases, bacteria, viruses and biological substrates, among others [34,45]. The biosensor is fabricated based on any one of the three previously highlighted formats: suspension in TEM grids, sandwich and microdroplet formats. Regardless of which format is used, the biosensor is usually composed of the substrate on which the LC molecules are applied (usually doped with some assembly molecules), while analyte recognition/interaction molecule may also be added. The ordering or molecular orientation of the LCs usually changes when the analyte of interest is added and interacts with the recognition/interaction molecules and this is the basis of the LC-based biosensing method. As mentioned in the previous section, the LC component of these sensors acts as signal amplifiers such that signals can be visually detected as a POM image. It must be mentioned that a few previous reports have attempted to improve the results delivered by LC-based biosensors. For instance, Iglesias et al. used a mixture of rod-shape and bent-shape mesogens with three different arrangement layers of OTS, a polyimide (PI) and a bent-core layer of amphiphilic molecules (Z4), and by factoring in the anchoring energy as a key determinant of LC response, they were able to widen the detection range of the sensor [85]. Furthermore, Vahedi and Kouchi recently simulated an easy fabrication of liquid crystal-based surface plasmon resonance (LC-SPR) biosensor and they showed that the proposed biosensor is capable of efficient sensing of analytes by detecting the change in the alignment of the LC [86]. Different forms of LC-based sensors that have been developed for biosensing are individually discussed in the following sub-sections.

### 2.1. LC-Based Glucose Sensors

Glucose is the main source of energy for the proper function of cells and organisms [36]. It is an important sugar whose level is maintained through specific mechanisms [21]. The normal level of glucose in the blood ranges from 3.6–6 mM [36], and this normal range is important for proper body function. A blood glucose level (BGL) higher than 11 mM leads to a condition called hyperglycemia, which causes various complications such as blurred vision, extreme thirst and slow wound healing [21,36]. These symptoms may not be noticeable until the BGL reaches a life-threatening level of 15–20 mM. An opposite condition of hypoglycemia refers to abnormally low BGL, which can also produce several complications such as seizures, neuroglycopenia and permanent brain damage. All these complications can be avoided if hypoglycemia and hyperglycemia are identified early enough. Therefore, it is important to detect BGL before it reaches either of the two extreme life-threatening levels. Over the last two decades, there have been numerous reports of LCs being used for the development of label-free glucose biosensors. These sensors lack the challenges experienced with mainstream diagnostic methods that are based on spectroscopy and electrochemistry such as ion interference, high cost and slow response time. LC-based glucose sensors are robust, cheap, selective and easy to operate. The LCs act as sensitive material for signal amplification such that the signal generated from the sensor can be visualized. 

**Figure 3:**
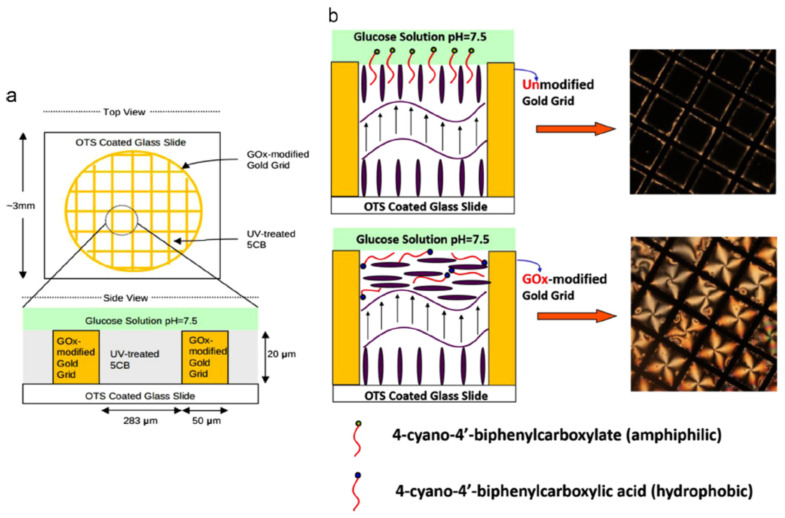
Schematic of enzymatic glucose sensor showing the gold grid on OTS-treated glass slide (**a**). The sensing mechanism (**b**): pH is maintained at 7.5 in the absence of GOx and so no changes in H^+^ concentration and dark image is obtained (top panel); with GOx-modified gold grid, glucose is oxidized and increased H^+^ concentration triggers a homeotropic to planar orientation of the LCs due to protonation of carboxylate ion. Reprinted with permission from ref. [48]. 2014 Elsevier.

The most widely used glucose detection principle is the oxidation of glucose in the presence of glucose oxidase (GOx) [21,22,36], though glucose detection has also been implemented by means of LC droplets [36]. The enzymatic reaction of glucose oxidase and glucose produces gluconic acid and hydrogen peroxide. Thus, H^+^ ions are produced and the pH of the system is lowered. Consequently, certain pH-responsive LC-based sensors can also be adapted for glucose sensing by monitoring the pH changes that accompany the action of glucose oxidase on glucose [81]. Fundamentally speaking, either or both pH and H_2_O_2_ can be monitored for the detection of glucose. In their glucose sensor development, Zhong and Jang reported a highly sensitive and selective LC-based sensor involving the use of UV-treated 5CB (4-cyano-4′-pentylbiphenyl) to produce amphiphilic 4-cyano-4′-biphenylcarboxylic acid (CBA) [48]. The resulting CBA means that UV-treated 5CB can exhibit pH-dependent optical signals. Therefore, in their experiment, they immobilized GOx on a gold grid and then filled the mesh with UV-treated 5CB, followed by immersion in glucose solution. The H^+^ produced from the oxidation of glucose triggered an optical response of the LCs from dark to bright as observed under a polarized microscope. In the absence of glucose, however, a dark image is obtained for there is no glucose oxidation. They were able to achieve a detection limit of 1 pM glucose, which is three orders of magnitude lower than most currently available glucose sensors. The general scheme of glucose sensing in the presence of GOx is illustrated in Figure 3. Similarly, Qi et al. reported the fabrication of a LC-based sensing platform for the detection of glucose and H_2_O_2_ [49]. Single-stranded DNA (ssDNA) was adsorbed onto the surface of nanoceria (CeO_2_) and the ssDNA gets dislodged from the surface in the presence of H_2_O_2_. Thus, the oxidation of glucose by GOx to produce H_2_O_2_ would dislodge the ssDNA from the surface of nanoceria thereby changing the alignment of 5CB from dark to bright. When glucose is absent, no H_2_O_2_ is produced and 5CB retains its homeotropic alignment at the LC/aqueous interface. This sensor was able to detect both H_2_O_2_ and glucose. Potentially, it can also detect any biomarker that depends on H_2_O_2_ concentration. Furthermore, Khan and Park functionalized 5CB with polyacrylic acid (PAA) and mixed polymer brushes of poly(acrylicacid-b-4-cynobiphenyl-4-oxyundecylacrylate) (PAA-b-LCP; LCP stands for liquid crystal polymer) and quaternized poly(4-vinylpyridine-b-4-cynobi-phenyl-4-oxyundecylacrylate) (QP4VP-b-LCP) for glucose detection [46]. According to these authors, the PAA makes the LC–aqueous interphase pH-sensitive while the QP4VP-b-LCP brush immobilized GOx without using coupling agents. The presence of glucose triggers a homeotropic to planar reorientation of the 5CB molecules, giving rise to dark to bright POM image transition accordingly. This sensor has a response linearity between 0.5–11 mM glucose and so it is suitable for BGL monitoring. These authors have also reported a glucose biosensor that uses a backscattering interferometry that gave a detection limit of 0.008 mM [87].

It should be pointed out that LC-based non-enzymatic glucose sensing has also been reported [37]. For instance, 3-Aminophenyl boronic acid (APBA)-decorated 4-cyano-4′-pentylbiphenyl (5CB) microdroplets have been used for non-enzymatic glucose sensing [37]. The binding between glucose and the APBA moiety on 5CB translated into reorientation of the 5CB droplets from radial to bipolar configuration. Such a change in configuration was absent when glucose was not present. This sensor was stable for up to 30 days and it presented high selectivity even in complex serum samples. The sensor gave a detection limit of 50 µM glucose, however, the limit of linearity was not established and so it is unclear if this sensor will be suitable for glucose monitoring in the normal BGL of 3.6–6 mM. Similarly, Ailincai and Marin also reported a non-enzymatic LC-based glucose sensor [50]. They fabricated a bio-responsive polymer-dispersed LC sensor for glucose and other bio-analytes. By using polymer-dispersed liquid crystal (PDLC) composites prepared by the encapsulation of cholesteryl acetate (L-ChAc) in polyvinyl alcohol boric acid (PVAB), they obtained selective responsiveness of the PDLC to sugars, amino acids and DNA. This was orchestrated by the fact that PVAB produced a uniform distribution of the cholesteric LC as micrometric droplets with moderate wettability. When blood glucose came in contact with the round droplets, the droplets disappeared leaving behind a “chicken-skin texture with rare light spots”, indicating weaker homeotropic alignment, which is attributable to new H-bonds forming between the OH group of glucose and the COO^−^ group of L-ChAc. LC membranes have also been used for the enhancement of amperometric glucose detection [47], though this sensor is not directly based on interfacial LC-alignment changes that give rise to a homeotropic to planar alignment and POM images. Overall, it is expected that LC-based sensors will continue to dominate the glucose sensing field for the foreseeable future.

### 2.2. Detection of Proteins, Peptides and Nucleic Acids

Proteins regulate vital processes in living systems such as immune responses and cell signalling [22,88,89]. Their detection is important in understanding their mode of action and working mechanism. Thus, accurate detection of proteins is also essential for identifying abnormal protein synthesis, which can signal the early stages of diseases. Similar to glucose, there have been several instances where LC-based sensors have been developed for protein detection and on the fundamental level, the composition of the sensor is similar to those of glucose in the sense that a specific functionality within the sensor is involved in some form of interaction with the target protein analyte thereby triggering a realignment of the LC molecules in the system, leading to the transformation of the homeotropic to the planar arrangement or vice versa [22].

**Figure 4:**
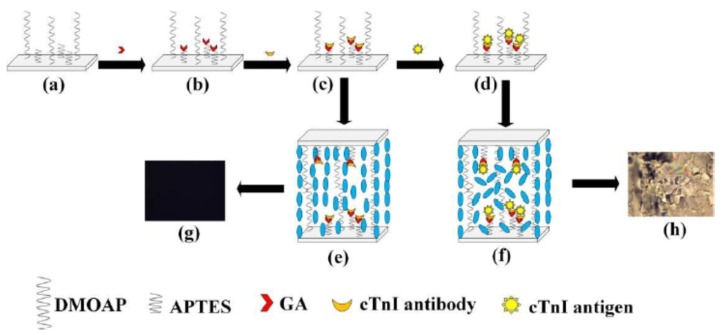
Schematic showing a protein (cTnI) LC-based sensor involving 5CB in a sandwich format. (**a**) Treatment of glass slide with DMOAP/APTES); (**b**) addition of crosslinking agent, GA; (**c**) immobilization of CnTI antibody; (**d**) reaction with CnTI antigen; (**e**) homeotropic LC alignment due to step (**c**); (**f**) antigen biding disrupts the alignment of LC molecues; (**g**) dark image due to step (**e**); and (**h**) bright image due to step (**f**). Reprinted with permission from ref. [51]. 2020 The Royal Society of Chemistry.

Very recently, Xia et al. reported the use of a new immunosensor based on 5CB liquid crystal for the detection of cardiac troponin I (cTnI), which is a protein that regulates the binding of myosin to actin [51]. They developed this LC-based sensor due to the challenges accompanying the use of established detection methods. By tethering cTnI antibody by means of glutaraldehyde (GA) to the surface of glass slides treated with *N*,*N*-dimethyl-*N*-octadecyl (3-aminopropyl) trimethoxysilyl chloride (DMOAP) and (3-aminopropyl) triethoxysilane (APTES), 5CB heated to isotropic phase was then sandwiched between two functionalized glass slides where these LC molecules adopt a homeotropic alignment induced by DMOAP/APTES. This gives rise to a dark image when observed in polarized light (Figure 4). However, if the target cTnI is present, it interacts with the cTnI antibody, thereby triggering a homeotropic to planar realignment of the LC molecules, giving rise to a bright POM image. This immunosensor presented a detection limit of 1 pg/mL, hence of high sensitivity, in addition to being low cost and of high specificity.

Using 5CB decorated with a nonionic surfactant dodecyl β-D-glucopyranoside, Wang et al. recently developed a LC-sensor for the detection of bovine serum albumin (BSA), concanavalin A (Con A) and lysozyme [90]. 5CB was loaded onto gold grids placed on OTS-modified glass slides, an aqueous solution was then placed on the 5CB layer where the LC molecules assumed planar alignment at the LC/aqueous interface, hence a bright POM image results. When the nonionic surfactant was added to the aqueous layer, it caused a realignment of the LC molecules to homeotropic where a dark POM image was obtained. This happens due to the formation of a stable self-assembled monolayer of the surfactant. Furthermore, the POM image changed back to bright when each of BSA, Con A and lysozyme was added to the biosensor. Detection limits of 0.001, 0.01 and 0.1 µg/mL were obtained for BSA, Con A and lysozyme, respectively, with this biosensor. Similarly, by monitoring the interaction between sodium polystyrene sulfonate (PSSNa) and a positively charged moiety coated on 5CB placed in a TEM grid cell, Omer and co-workers developed a LC-based biosensor for the detection of BSA, α chymotrypsinogen-A (ChTg) haemoglobin (Hb) and lysozyme [91]. Homeotropic orientation of the 5CB in the TEM grid cell changed to planar orientation when the protein solutions were injected into the cell. The same research group has also reported a similar protein and DNA biosensor based on a similar principle of fabrication [92]. This shows again that it is the interaction between an analyte and recognition molecule in the sensor that triggers reorientation of the LC molecules at the LC/aqueous interface giving rise to visually detectable bright or dark POM image. Ligand/receptor detection is important for analyte and drug screening. Therefore, a LC-based sensor has been reported for detecting avidin-biotin specific binding [93]. Using a microfluidic approach, a LC-based droplet biosensor was fabricated by functionalizing 5CB droplets with PAA-b-LCP (poly(acrylicacid-b-4-cynobiphenyl-4′-undecylacrylate)) with the PAA chains on the LC molecules biotinylated and used for the detection of avidin-biotin binding at the LC/aqueous interface. This binding leads to a configurational change of the LC droplets from radial to bipolar. This biosensor can detect avidin as low as 0.5 g/mL and it can discriminate between the avidin target analyte against other proteins such as BSA, Hb, lysozyme and chymotrypsinogen. Popov et al. has also demonstrated the use of a LC-based biosensor for specific detection of goat Immunoglobulin G (IgG) antigen [65]. 5CB molecules were coated with biotinylated lipids and biotinylated anti-goat IgG. When the analyte goat IgG was applied to the functionalized LC molecules in the TEM grid cell, there was a homeotropic to planar transition that gave rise to dark–bright transition in the POM image. This biosensor did not respond to negative controls of rat or rabbit serum IgG, thus proving the specificity of the sensor for goat IgG. Ren and Jang have also reported a LC-based aptasensor for the detection of the clinically important carcinoembryonic antigen [66]. The analyte binds to a specific aptamer immobilized on the surface of modified glass slides, triggering a reorientation of the 5CB molecules from homeotropic to random alignment (i.e., nematic domains with randomly oriented optical axes). The biosensor was able to discriminate between the analyte and BSA and human serum albumin (HSA).

Various cationic gemini surfactants were used to decorate the LC/aqueous interface in a LC-based protein sensor [94]. The sensor initially produced a dark POM image due to the stable monolayer of surfactants formed at the interface. This dark image then changed to a bright image upon addition of BSA, which is a negatively charged protein in a neutral environment, triggering a realignment of the LC molecules from homeotropic to planar. In a similar vein, Verma et al. has recently reported the use of a LC-based sensor for the identification of the secondary structure of Cyto, BSA, Hb, Con A and fibronectin (FibN) [95]. They used surfactin, a cyclic lipopeptide, to promote the homeotropic alignment of LC molecules at the LC/aqueous interface to produce a dark POM image initially. However, the colour changed from dark to bright in the presence of nanomolar concentrations of Cyto, BSA, Hb, Con A and FibN at neutral pH, which was interpreted on the basis of interaction between the anionic headgroups of surfactin and the proteins. The specific patterns observed in the bright POM image is determined by the specific form of interaction between the proteins and surfactin. Wu and coworkers have reported the use of dye liquid crystals (DLC), which exhibits both optical anisotropy and dichroic absorption for the quantification of BSA [52]. The DLC consists of an azobenzene liquid crystal molecule that has been modified with two azo groups to serve as the chromophore. They exploited the dichroic absorption of azobenzene at 470 nm for the transmission spectrometric determination of BSA concentration while using the birefringence characteristics of the LC as the trigger for the transmission intensity change as BSA concentrations varied. 

In a somewhat different dimension, a research group has reported the use of LC-based sensors for imaging microcontact printed proteins [56]. A homeotropic to planar transition of the LC confirms the specific binding between a target anti-biotin IgG and biotinylated BSA while such transition does not take place when a control anti-goat IgG was used. This sensor may form the basis for fabricating functional surfaces on which affinity microcontact printed proteins can be imaged. Su et al. reported a LC-based immunosensor for detecting human β-defensin-2 (HBD-2), a cysteine-rich cationic peptide with antimicrobial activity [55]. In this sensor, 5CB was sandwiched between two glass slides whose surfaces had been suitably treated with DMOAP/APTES followed by the addition of HBD-2. When the anti-HBD-2 antibody was then applied, the alignment of the 5CB molecules changed from homeotropic orientation to randomly oriented nematic domains, giving rise to a dark to bright POM image transition that is visually detectable in polarized light. The sensor gave a linear concentration dependence in the range 1–10 ng/mL with a limit of quantitation of 0.53 ng/mL.

Deoxyribonucleic acid (DNA) is the fundamental basis of life and its damage or mutation can lead to lethal consequences. Ribonucleic acid (RNA) is also important in the regulation of various biological processes and changes in the concentration of certain non-coding RNA may be an indicator for disease onset. Therefore, reliable sequence-specific detection of DNA and RNA is important. To this end, several reports have presented the use of LC-based sensors for DNA detection. For example, a research group has presented a highly sensitive LC-based sensor for the detection of p53 mutation gene segment using a dendrimer-mediated approach [96]. Mutation in the p53 gene may signal several diseased states such as brain tumour and liver disease. 5CB doped with SDS was applied to a copper grid cell placed on a DMOAP-coated glass slide. DNA dendrimers applied to the grid created a tilted alignment of the LC molecules at the LC/aqueous interface. However, when the mutant-type target was added, its interaction with the DNA dendrimers induced the rearrangement of the LC molecules from tilted to homeotropic alignment. The target can be detected in the 0.08 to 8 nM concentration regime with high sensitivity. Liu and Yang also reported a LC sensor for the multiplex detection of DNA [63]. 5CB was drawn into the space between two glass slides functionalized by droplets of DNA or PNA (peptide nucleic acid) solutions. The slides were immersed in NaCl solution or other sodium salt solutions such that the sodium ion would complex with the negatively-charged DNA backbone but not with the neutral PNA backbone. The PNA-containing system has the LC molecules in homeotropic alignment so that when a Cy3-DNA target was applied, the interaction between this target and PNA triggers realignment of the LC molecules giving a POM image transition from dark to bright. A limit of detection of 10 fM was reported. Furthermore, Kim and Jang reported the use of a LC-based sensor for the detection of single-strand breaks (SSBs) in DNA [64]. SSBs lead to carcinogenesis and ageing, and so their detection is crucial to human well-being. A single-stranded DNA (ssDNA) adsorbed onto the cationic surfactant (OTAB) present in the LC/aqueous interface formed by immersing 5CB-filled copper grids in the OTAB solution induces a planar orientation of the LC molecules, giving a bright optical image in polarized light. However, a DNA consisting of SSBs would give a decreased electrostatic interaction with the cationic surfactant, thereby causing a rearrangement of the LC molecules to homeotropic alignment, giving a dark POM image. This sensor makes the detection of SSBs in DNA easier to implement.

### 2.3. Detection of Disease Biomarkers

**Figure 5:**
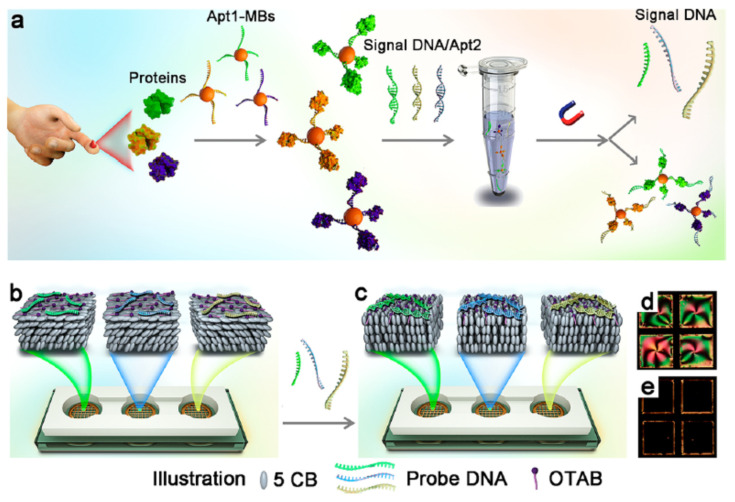
Schematic illustrating the simultaneous detection of multiple tumour biomarkers in blood. (**a**) Tumor markers are captured by apt1-coated magnetic beads, triggering the release of signal DNA after incubation with signal DNA/apt2 duplexes. (**b**) Planar orientation of LCs prior to hybridiza-tion. (**c**) Homeotropic alignment at the aqueous/LC interface due to specific hybridization of signal DNA to each corresponding probe DNA. (**d**) Bright POM imgae corresponding to (**b**). (**e**) Dark appearance corresponding to (**c**). Reprinted with permission from ref. [67]. 2020 American Chemical Society.

LC-based sensors have also been developed for the detection of various disease biomarkers. The principle upon which these kinds of sensors are based is similar to those of proteins and peptides discussed in the last subsection; a molecule that is capable of interacting with the target biomarker is incorporated into the sensor. The interaction that takes place in the presence of the biomarker is what triggers an orientational or configurational change of the LC molecules. Kim and Jang recently presented a LC-based aptasensor for detecting interferon-γ (IFN-γ), a cytokine produced in T-cells [97]. Elevation of this protein may indicate the onset of various diseases such as tuberculosis (TB). An aptamer immobilized on the surface of a DMOAP/APTES-treated glass slide consisting of 5CB molecules binds IFN-γ and thereby disrupts the alignment of the LC molecules, changing the POM image from dark to bright. The biosensor was able to detect this disease biomarker at 17 pg/mL. In addition, the sensor showed capability for the diagnosis of TB in blood samples of TB patients. In principle, this biosensor can be used for the diagnosis of other diseases for which IFN-γ has been implicated. Similarly, these authors reported another LC-based biosensing platform that made use of polymeric surfaces for the detection of anti-TB antibodies in solution [98]. Qi et al. also recently reported a new LC-based biosensor for simultaneous detection of multiple tumour biomarkers in blood [67]. In this work, the researchers used aptamer-coated magnetic beads to capture target proteins in blood followed by incubation with the duplex of another aptamer (apt2) and a signal DNA. The presence of target protein in the blood sample induces the release of signal DNA from the duplex into the aqueous solution, where the signal DNA hybridizes with a complementary probe DNA present with 5CB molecules. This causes the orientation of the LC to change from planar to homeotropic alignment at the LC/aqueous interface, producing a bright to dark transition. Figure 5 presents an illustration of the detection scheme. The sensor was shown to be suitable for the simultaneous detection of several tumour markers such as prostate specific antigen (PSA), carcinoembryonic antigen (CEA) and alpha-fetoprotein (AFP) with high specificity and sensitivity. In a related development, Ding et al. reported a biosensor for in vitro detection of human cancer cells, SK-BR3 using a LC-based sensor consisting of 5CB droplets functionalized with Herceptin antibody [99]. The conjugated LC molecules in the microdroplets displayed orientational change from radial to bipolar when the target SK-BR3 breast cancer cells were present. The microdroplets were selective and discriminated against controls of 10% human plasma, KB cancer cells and fibroblast. 

### 2.4. Detection of Urea, Urease and Other Enzymes

Urea as the main metabolic product of protein metabolism is produced as a result of the decomposition of nucleic acids and proteins [58]. It is an important indicator for clinical analysis and diagnosis. For instance, the concentration of urea in the blood may exceed the normal range of 155–390 mM, leading to several complications such as urinary tract obstruction with shock, burns, renal failure and gastrointestinal bleeding (this is within the normal range of urea in urine). Therefore, reliable and sensitive techniques for urea detection are important. Khan and coworkers reported a LC-based urea biosensor using a 5CB-filled TEM grid on an OTS-coated glass substrate [58]. By placing PAA-b-LCP in the LC/aqueous interface and covalently linking urease to the PAA chains, urea was detected as reported by the transition from planar orientation to homeotropic alignment when the sensor was observed under a polarized microscope. The interaction between urease and urea created a change in pH due to the production of ammonia, which leads to a conformational change in the PAA chains. Using the same chemical composition in LC droplets format, this same research group was able to detect urea at concentrations as low as 3 mM [57]. Duan et al. also reported a sensitive and quantitative LC-based biosensor for urea detection in droplet format [35].

Hu and Jang have developed a LC-based sensor for real-time detection of urease [61]. The working mechanism of this sensor is similar to that previously described for urea detection in the sense that the ammonia produced via urea hydrolysis by urease changes the pH of the system, triggering an orientational transition of the LC molecules. In this case, UV treatment of 5CB produced CBA, which occupies the LC/aqueous interface. The presence of urease is indicated by the production of ammonia, which changes the pH thereby deprotonating the CBA and causing a planar to homeotropic alignment of the LC molecules. When a Cu(II) urease inhibitor was present, the planar to homeotropic transition did not take place. This sensor can easily monitor 1 nM urease in real-time. A similar approach was used by Qi et al. to simultaneously detect urease activity and heavy metal ions [100]. This is a smart approach in the sense that the inhibitory effect of heavy metal ions which produces the opposite POM image in urease sensing was simply used as a signal for heavy metal ion detection. Using a LC droplet format, Liu and Jang have also reported a label-free approach for imaging urease activity [101]. The droplet patterns were formed spontaneously by spreading an organic solution of 5CB doped with stearic acid on microscope slides. The droplets showed a bright POM image in the presence of urea or urease solution but a dark image in the presence of both urea and urease, indicating that the mixture produces ammonia, which changes the pH and deprotonates the stearic acid, leading to an orientational transition of the LC molecules in the droplet.

The pancreas produces a digestive enzyme called trypsin, which maintains the function of the digestive system in humans by hydrolyzing proteins into peptide fragments for easy transport into the small intestine for use in cellular metabolism [53]. Trypsin is also responsible for blood coagulation and immune response. It also plays a major role in catalyzing the hydrolysis of peptide bonds [54]. Hu and Jang have reported a LC-based sensor for imaging trypsin activity [102]. They added a solution of poly-L-lysine (PLL) to a self-assembled monolayer of a phospholipid, DOPG (dioleoyl-sn-glycero-3-phospho-rac-(1-glycerol) sodium salt) present at the LC/aqueous interface of the sensor. Electrostatic interaction between the positively-charged PLL and negatively-charged DOPG triggers an orientational transition of the LC molecules from homeotropic to planar orientation (i.e., dark to bright image). When trypsin is added to the system, it catalyzes the hydrolysis of PLL, giving rise to a bright to dark transition in the POM image. This means that when a mixture of PLL and trypsin would be added to the DOPG-decorated LC interface, there would be an orientational transition from homeotropic to planar state. Wang et al. reported a LC-based sensor for the detection of trypsin using BSA as the enzyme substrate and dodecyl trimethyl ammonium bromide (DTAB) as the surfactant for inducing the alignment of LC molecules [54]. Adding BSA to the self-assembled monolayer of DTAB at the LC/aqueous interface induces the orientational transition of the LC molecules such that dark to bright transition takes place. However, the dark image persists in the presence of trypsin and BSA due to hydrolysis of the BSA by trypsin. This sensor was able to detect as low as 0.1 U/mL of trypsin. Similarly, hydrolysis of BSA by trypsin has been experimented for sensing trypsin in a LC-based sensor whereby peptide fragments resulting from the hydrolysis disrupted the orientation of 5CB molecules at the LC/aqueous interface leading to a dark to bright transition [53].

In the development of a LC-based biosensor for detection of acetylcholinesterase (AChE) and its inhibitor, Wang et al. used a cationic surfactant, myristoylcholine chloride (Myr) to functionalize 5CB. The LC/aqueous interface was disturbed in the presence of AChE leading to a dark to bright optical change [60]. Furthermore, a previous publication reported a LC-based sensor for monitoring lipase activity [59]. The sensor consists of 5CB doped with glyceryl trioleate, which can be hydrolyzed enzymatically by lipase. The resulting oleic acid is capable of forming a self-assembled monolayer at the LC/aqueous interface thereby changing the alignment of the LC molecules from planar to homeotropic state (bright to dark optical change). This transition was not observed in the absence of glyceride. Similarly, a LC-based biosensor was developed and used to monitor cell viability by way of monitoring lipase activity since members of this enzyme family are released in the course of cell necrosis [103]. The sensor consists of 5CB and a monolayer of phospholipids. Therefore, in the presence of small amounts of phospholipases, which hydrolyze phospholipids, the LC-lipid interface is disrupted causing an orientational transition of the LC from homeotropic to planar state. This sensor was found to be superior in performance compared to a fluorescence assay. Zhou and coworkers also reported a sensitive and label-free LC-based detection system for carboxylesterase, a biomarker for hepatoma cells [62], whose quick dark to bright optical response gave a detection limit of 18 U/L for this enzyme. In the same vein, a LC-based pH sensor was used to monitor enzymatic reactions in penicillinase [104], just as a similar sensor has been reported for label-free detection of catalase [105]. Furthermore, a LC-based sensor has been reported for detecting cellulase and cysteine [106]. When 5CB was functionalized with a surfactant dodecyl β-d-glucopyranoside, the cellulose enzymatically hydrolyzed this surfactant, giving rise to an orientational transition of the LC molecules (dark to bright transition). This transition also occurs in the presence of cysteine and Cu(II) as cellulase is also able to hydrolyze the surfactant when Cu(II) is present due to the strong biding between cysteine and Cu(II). The sensor recorded a detection limit of 1 × 10^−5^ mg/mL and 82.5 µM for cellulose and cysteine, respectively. LC-based biosensors have also been reported for the detection of thrombin, an effector protease of the coagulation cascade involving conversion of circulating fibrinogen to fibrin monomers [107,108], where disruption of a self-assembled monolayer of surfactant at the LC/aqueous interface induces orientational transitions of the LC molecules, reporting a visual transition from dark to bright or vice versa.

### 2.5. Detection of Bile Acids and Other Physiologically-Important Analytes

Cholic acid is one of the bile acids synthesized in the liver, so it represents a biomarker for liver and intestinal diseases. Cholic acid constitutes about 31% of bile acids [109,110]. He and coworkers have reported a LC-based biosensor for the detection of this acid using 5CB-filled TEM grids supported on a coated glass substrate and surfactants such as SDS, C12E4 and DTAB [109]. The surfactant molecules assemble at the LC/aqueous interface inducing homeotropic alignment of the 5CB molecules. When cholic acid is present, it disrupts this alignment due to competitive adsorption of the acid at the LC/aqueous interface thereby producing orientational transition of the LC molecules from homeotropic to planar alignment. They found that the detection limit, which varied from 12–200 µM was dependent on the pH and ionic strength of the aqueous phase and the head group of the surfactants [109]. Using LC droplets consisting of SDS surfactant, Niu et al. also developed a biosensor for cholic acid [110]. This biosensor shares similar characteristics with that of He and coworkers and the mechanism of sensing is also similar. They observed a radial to bipolar transition due to competitive adsorption of the cholic acid at the LC/aqueous interface. They reported a detection limit of 5 µM, which is lower than that found by He and coworkers. It must be mentioned here that some research groups have developed biosensors for the detection of total bile acids rather than cholic acid alone [111,112], and the mechanism of detection is similar to those two previously described except that the analyte here is bile acids. Specifically, Deng and coworkers used microfluidic channels for their sensor and functionalized the 5CB droplets with sulfated β-CD/tetradecyl sulfate sodium (SC14S) complexes and poly(diallyldimethylammonium chloride) (PDADMAC) [112]. They also reported that the limit of detection can be tuned based on the number density of the LC droplets.

Monitoring cholesterol levels in humans is of great importance due to its association with cardiovascular diseases. Therefore, several sensing protocols are available for its detection, including the recent development of LC-based biosensors [113,114,115]. Munir et al. reported a 5CB sensor where 5CB placed in TEM grid was coated with PAA-b-LCP, followed by immobilization of cholesterol oxidase and horseradish peroxidase on the PAA chains. The presence of cholesterol was confirmed by the orientational transition of the LC molecules from planar to homeotropic arrangement [113]. In their case, Wei and Jang developed a pH-responsive LC-based biosensor for cholesterol detection [114]. They reported a stable sensor that performed well in the physiologically-relevant cholesterol concentration range of 90–220 mg/mL with a detection limit of 1 pg/mL.

### 2.6. Detection of Whole Cells, Microorganisms and Other Analytes

LC-based sensors have also been reported for biological cells and microorganisms. Han et al. reported the detection of virus cells on a polymeric surface with periodic nanostructures [70]. They immobilized human cytomegalovirus antibodies and adenovirus antibodies onto a gold-coated polymeric surface and observed uniform orientation of the 5CB molecules. However, when the target human cytomegalovirus and adenovirus was each added, it induced a change in the orientation of the LC molecules to a random state (i.e., nematic domains with randomly oriented optical axes). They used this orientational transition to sense these viruses and confirmed the specificity of the binding events with atomic force microscopy (AFM) and ellipsometry. Furthermore, Zafiu, et al. reported a LC-based sensor for bacterial detection [116]. Lipopolyssacharides were applied on top of a 5CB-filled gold grid and produced a homeotropic alignment, which was disrupted in the presence of different bacteria species, irrespective of their viability. In a related experiment, Das and coworkers also reported using a LC-based sensor for recognition of the interactions between cell membrane components and bacterial endotoxin by using the optical transition of the 5CB molecules present in the gold grid of the sensor [117]. Similarly, a LC-based sensor has been reported for monitoring the antibacterial activity of *E. coli*, using chitosan-reinforced graphene oxide (GO), which is capable of rupturing phospholipids [118]. Fang et al. has used 5CB as an optical amplification medium for imaging neurons, fat cells and muscle cells [69]. They applied 5CB on a cell-covered glass slide and a poly-d-lysine coated glass slide was placed atop the 5CB to create a sandwich structure. According to these authors, preferential orientation of the LC molecules on the cell surface produced well-resolved images. Furthermore, a LC-based biosensor has been used for the detection of a plant pathogen, root-knot nematodes (*Meloidogyne* species) [119]. Plant pathogens are the main culprits responsible for lower crop yields, so their detection is of great importance in food safety and quality. Parveen and Prakash used the orientational transition observed in the LC molecules to sense plant pathogens.

**Scheme 2:**
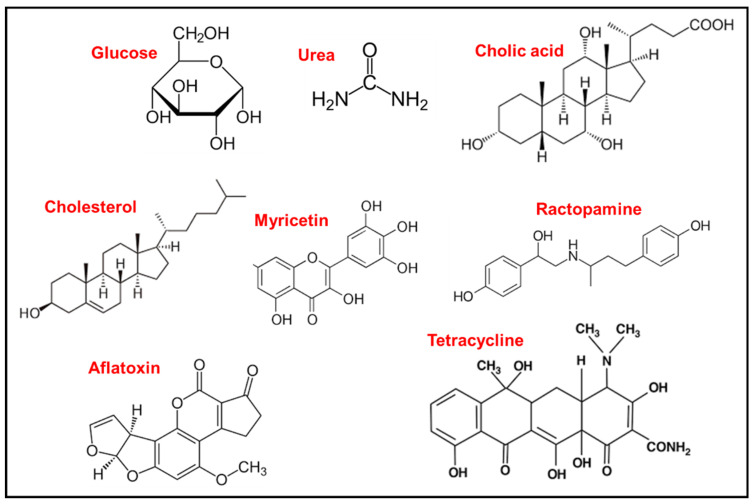
Chemical structures of selected analytes in this section.

LC-based sensors have also been reported for myricetin, a naturally-occurring flavonoid. It is known to accelerate the degradation of single-stranded DNA (ssDNA) and double-stranded DNA (dsDNA) and has chemotherapeutic properties [120]. Using a 5CB-filled TEM grid cell functionalized with DTAB followed by spontaneous adsorption of DNA, Munir and Park developed a LC-based sensor for myricetin [120]. DNA adsorption changed the LC alignment from homeotropic to planar state due to electrostatic interaction between DNA and DTAB. However, when myricetin was injected into the TEM grid cell, planar orientation of the LC molecules changed to homeotropic alignment due to degradation of the DNA by myricetin. It was shown that the sensitivity of this sensor to myricetin was enhanced by metallic salts of Fe and Cu. A LC-based imaging sensor was also reported for ractopamine, a synthetic β-andrenergic agonist [121]. Using gold nanoparticles for signal enhancement, Wang et al. immobilized ractopamine aptamers on a treated glass slide and 5CB molecules were applied and a second treated glass slide was placed on top, making a sandwich arrangement. Gold nanoparticles with ractopamine adsorbed on them were then applied and this caused an orientational transition of the LC molecules from homeotropic to planar state, giving a dark to bright POM image transition. This sensor gave a ractopamine detection limit of 1 pM. Furthermore, a LC-based immunosensor was reported for the detection of aflatoxin, a toxic mycotoxin that is capable of causing several health problems [122]. This sensor used BSA-aflatoxin on the DMOAP/APTES-treated glass slide and 5CB was applied followed by aflatoxin antibody, giving a disordered arrangement of the LC molecules (i.e., nematic domains with randomly oriented optical axes). When target aflatoxin was added, however, competitive binding between the analyte and the BSA-aflatoxin conjugate for the antibody disrupts the LC orientation to give a homeotropic alignment. This sensor presented high specificity and a detection limit of 100 pg/mL. Similarly, a LC-based aptasensor has been reported for the detection of tetracycline, a broad-spectrum antibiotic [123]. 5CB-filled copper grid was placed on a DMOAP/APTES treated glass substrate that also contained adsorbed aptamer molecules, followed by another treated glass slide on top, giving a sandwich arrangement. In the absence of tetracycline, the LC molecules assume homeotropic alignment (dark POM image) while the addition of tetracycline disrupts the LC alignment due to interaction with the aptamer, thereby giving a bright POM image. A summary of all the sensors discussed in this section is presented in Table 1. Furthermore, Scheme 2 shows the chemical structures of selected analytes discussed in this section.

## 3. LC-Based Sensors for Heavy Metal Ions, Nitrite and pH

### 3.1. Detection of Heavy Metals

Heavy metals are ubiquitous and constitute major contaminants in water and the environment [21,72,124]. Their ions can easily form bonds with protein functional groups, so they can cause serious health problems upon entering the cell [21]. Therefore, accurate detection of heavy metals is crucial for a clean and safe environment and for human well-being. Most well-established methods for heavy metal detection are sophisticated, expensive and require a skilled operator. LC-based sensors offer a simple, easy and low-cost approach to heavy metal detection with reasonable specificity and sensitivity.

**Figure 6:**
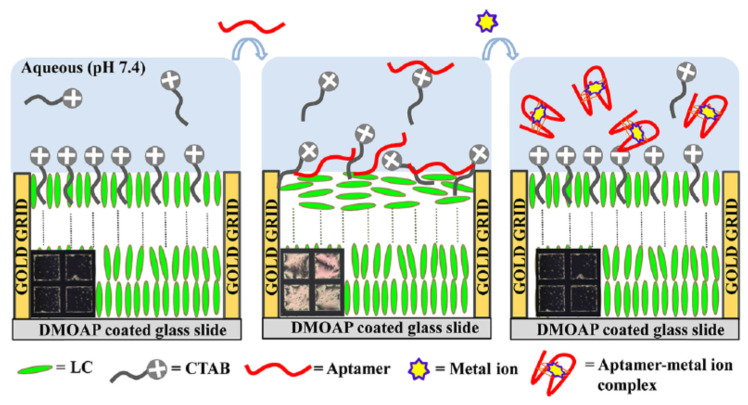
Schematic showing orientation of 5CB molecules at the LC/aqueous interface. Reprinted with permission from ref. [73]. 2019 American Chemical Society.

Using UV-treated 5CB confined in urease-modified gold grid immersed in urea solution to produce ammonium and hydroxide ions, Hu and Jang used Cu(II) as a model heavy metal ion to inhibit the action of urease on urea and thereby keeping the original orientation of the LC [124]. However, they observed a planar to homeotropic transition in the absence of Cu(II). Han and Jang also reported a LC-based heavy metal ion sensor using stearic acid-doped 5CB droplet patterns spontaneously generated on an OTS-coated glass substrate by evaporation of a heptane solution of the LC in the nematic state [72]. At a pH of 8.1, the heavy metal ions of copper (II) and cobalt (II) interacted with the deprotonated stearic acid thereby disrupting self-assembled acid at the LC-aqueous interface and giving a dark to bright transition of the POM image. Furthermore, Du et al. reported a LC-based sensor for the detection of lead (II) ions using aggregation-induced emission (AIE) luminogens [71]. Detection is based on variation in the fluorescence intensity generated by disrupting the LC alignment at the LC/aqueous interface when Pb(II) ions were present. This disruption is caused by a Pb(II)-specific DNAzyme incorporated on DMOAP/APTES treated glass substrate and it gave a fairly low detection limit of 0.65 nM compared to other similar Pb(II) detection methods. Similarly, Verma and coworkers developed a LC-based Pb(II) sensor on the basis of an aptamer-target binding event [73]. They used spinach RNA as a recognition probe. Using a cationic surfactant of CTAB and spinach RNA aptamer, there is an orientational transition of the 5CB molecules at the LC/aqueous interface from the planar to homeotropic state as a result of the formation of a more stable quadruplex structure of the RNA with Pb(II) in preference to the less stable CTAB-RNA complex (see Figure 6). This sensor offers a detection limit of 3 nM, which is about six times higher than that reported by Du and coworkers. Using surfactant-stabilized NiFeO_4_ nanoparticles, Zehra et al. reported a label-free LC-based sensor for Pb(II) [125]. They observed a homeotropic to planar transition for the LC molecules at the LC/aqueous interface in the presence of Pb(II), which interacts with abundant OH groups on the surface of the nanoparticles inducing a disruption of the LC alignment. They reported a detection limit of 100 ppb. Hong et al.used an aptamer as the recognition moiety in developing a Hg(II) ion LC-based sensor [126]. Using LC droplets consisting of OTAB surfactant with 5CB in the presence of an aptamer specific for Hg(II), they observed a planar to homeotropic orientational transition of the LC in the presence of the heavy metal ions induced by the weakening of OTAB-aptamer electrostatic interactions by the Hg(II) ions. In the same vein, Singh and coworkers developed a label-free Hg(II) sensor based on MeDTC-doped 5CB [27]. In the presence of Hg(II) ions, a dark to bright transition of the POM image was observed. This was due to a rapid complexation between the chelating group of MeDTC and Hg(II) ions, which disrupted the LC alignment at the LC/aqueous interface. A report has also presented the use of a LC-based sensor for the detection of calcium ions in water, whereby the PAA chains of PAA-b-LCP used to functionalize 5CB complexed with the metal ions, triggering homeotropic to planar transition [127].

### 3.2. Detection of Nitrite

While the nitrite ion (NO_2_^−^) is useful in several fields such as chemical and food industries and appear harmless, its conjugate acid, HNO_2_, is capable of reacting with secondary amines to form carcinogenic nitrosamines and it can also oxidize Fe(II) to Fe(III) in Hb thereby hampering the ability of Hb to carry oxygen [128]. Therefore, constant monitoring of nitrite is important. A LC-based nitrite sensor has been reported that uses diazotization reaction as the sensing mechanism [128]. This sensor consists of decylaniline-doped 5CB placed in a TEM grid on a glass substrate. The LC adopts a planar orientation when nitrite is absent. In the presence of nitrite, however, the nitrite ion reacts with decylaniline to yield diazonium ions and thus the LC undergoes orientational transition that gives rise to homeotropic alignment (bright to dark colour transition). This high selectivity nitrite sensor gives a detection limit of 25 µM and the author also used a built-in digital camera to record the optical transitions.

### 3.3. pH Sensors

pH is a measure of the hydronium ion concentration in a sample solution and its value is critical in various fields such as health, food, pharmaceuticals, environmental and life sciences [80]. Electrochemical pH measurement is the gold standard, but such may not be suitable for long-term monitoring due to its susceptibility to drifts. LC-based sensors are now being developed as pH sensors. Results from these sensors can be simply interpreted based on POM images. pH-responsive sensors can be used as biosensors where biological interactions are pH-dependent [81]. Chen and coworkers reported the development of LC-based pH sensor for continuous monitoring of pH in flow aqueous systems [80]. They doped 5CB molecules with various weak acids as pH responsive molecules and filled the TEM grid placed on a coated glass substrate with the doped 5CB. When the sensor is immersed in an aqueous solution, the dopants dissociate and align at the LC/aqueous interface due to changes in pH and disrupt the alignment of the LC molecules giving bright to dark image transition, which is observable by the naked eye. The reverse transition takes place if the pH of the aqueous solution decreased on immersing the sensor in it. They were able to adjust the pH range for the optical transition from 6.8 to 8.2 based on the dopant selection. They showed they could arrange four individual sensors each with a different dopant in a single device and used it to monitor pH level for drinking water from 6.5 to 8.5 with a 1 s response time. Similarly, using functionalized cholesteric LC (CLC) double emulsion droplets (DEDs), Jang and Park developed a LC-based sensor for pH monitoring [81]. They reported that by coating the CLC DEDs with PAA-b-LCP, they become pH-responsive due to deprotonation and protonation of the carboxylate on the PAA chain, leading to the planar and homeotropic orientation of the LC molecules, respectively. Table 2 presents a summary of all the sensors discussed in this section.

## 4. LC-Based Detection of Gases, VOC and Toxic Substances

### 4.1. Gas Sensors

**Figure 7:**
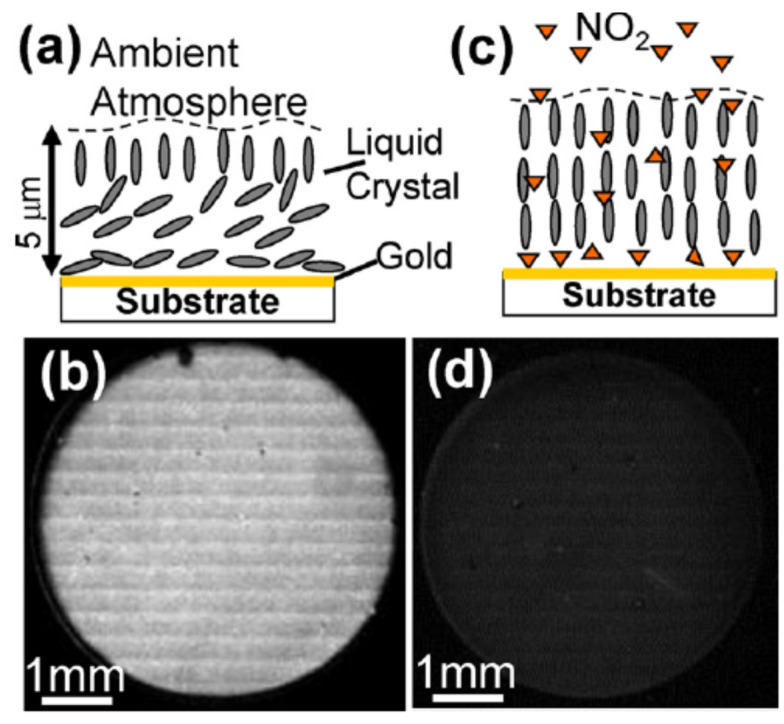
Illustration of the NO_2_ sensing mechanism. (**a**) Organization of LC molecules on a gold coated substrate; (**b**) bright image corresponding to (**a**) when viewed between crossed polarizers; (**c**) A change in the orientation of the LC due to binding of NO_2_ at the gold–LC interface; and (**d**) dark image corresponding to (**c**) when viewed between crossed polarizers. Reprinted with permission from ref. [77]. 2012 Elsevier.

Due to their simplicity, low cost and ease of fabrication, LC-based sensors are now appearing in the literature for the detection of various gaseous substances, including toxic gases. For instance, nitrogen oxide (NO_2_), a ubiquitous environmental pollutant is toxic and prolonged exposure to it can lead to death. A LC-based sensor has been reported for this gaseous pollutant by Sen and coworkers [77]. Using LC film supported on a gold-coated substrate, the sensor is able to adsorb NO_2_ and the transport of NO_2_ molecules to the gold surface induces the orientational transition of the LC molecules at the LC–gold interface (Figure 7). The sensor is selective for NO_2_ only while it does not respond to other atmospheric gases over the course of 200 s. Furthermore, a LC-based sensor has been reported for sensing carbon monoxide gas (CO) by using a LC doped with magnetite (Fe_3_O_4_) nanoparticles and intercalated into porous alumina (Al_2_O_3_) [79]. The interaction between CO and the nanoparticles dispersed in the LC is the basis for the sensing mechanism. According to the authors, this interaction causes a shift in the selective reflection peak wavelength, which is proportional to CO concentration, which they estimated to be 0.85 nm/(mg/m^3^) in air. A highly selective and sensitive optical sensor based on LC has also been reported for ammonia gas [129]. Using chitosan-Cu(II)-decorated glass substrate, the alignment of 5CB on the substrate is disrupted due to competitive binding between ammonia gas and Cu(II) on the glass substrate, thereby causing the orientational transition of the LC molecules from homeotropic to planar state. A detection range of 50–1250 ppm was reported for this ammonia sensor with a detection limit of about 17 ppm.

### 4.2. VOCs Sensors

Volatile organic compounds (VOCs) are organic compounds with fairly high vapour pressure such that they easily evaporate under ambient conditions [130]. They cause pollution to the environment and are harmful to humans. Their presence in air can also be used as a biomarker for diseases [131]. Therefore, sensing and monitoring VOC gases is important for our safety and well-being. To this end, Wang et al. recently reported a LC-based chemical sensor for toluene and acetone vapours [131]. They used LC/polymer composite fibres electrospun and spread out as a mat on a glass substrate. Absorption of these analyte vapours changes the optical properties of the LC/fibre mats producing sensitive and reversible detection. A report has also presented the use of a chiral-nematic LC encapsulated in microscale polyvinylpyrrolidone via electrospinning for gas sensing [78]. Similarly, a fibre-optic VOC gas sensor has been reported by Tang and coworkers [130]. They used a cholesteric LC film coated side polished fibre (CLCFC-SPF) to sense VOC gases such as acetone, methanol and tetrahydrofuran (THF). On applying light to the sensor, they observed selective wavelength coupling from the SPF to the CLCF that resulted in resonant dips in the transmitted spectrum, and the pitch of the CLCF increased as a function of VOC gas concentration. Similarly, using a LC sandwiched between two modified electrodes, Dadoenkova et al. has also reported a sensor that may be suitable for chemical vapour sensing [132]. It must be mentioned that another LC-based optical sensor was reported for the detection of butylamine vapour in air [133].

### 4.3. Detection of Toxic Substances

Substances that are toxic to plants and animals must be continuously monitored in the environment to safeguard our health and well-being. In that regard, several LC-based sensors have been reported for the detection of toxic substances. For instance, Hu and coworkers recently reported a LC sensing platform for selective detection of uranyl ion (UO_2_^2+^) [74]. The sensor is composed of a UO_2_^2+^–dependent DNAzyme, its substrate, a capture probe and 5CB sandwiched between two treated glass slides. In the presence of the analyte UO_2_^2+^, the DNAzyme cleaves the substrate at the rA site, and the cleaved product hybridizes with a capture probe to form a duplex, which disrupts the original alignment of the LC molecules. This gives a dark to bright transition. This specific and label-free sensing method gave a detection limit of 25 nM and this platform can be adapted for the detection of other radioactive substances.

Organophosphonates are highly toxic chemicals that are used as warfare agents [15]. Therefore, there is a strong interest in reliable methods of monitoring and reporting the presence of organophosphonates at low concentrations on battlefields. Wang et al. has reported a LC-based sensor for dimethyl methylphosphonate (DMMP) vapour [15]. They used 5CB films consisting of Cu(II) ions applied to functionalized substrates. When present, DMMP interacts with Cu(II) ion and this causes a disruption of the LC alignment from homeotropic to planar orientation. They reported a detection limit of 0.51 mg/m^3^. A similar experiment was reported by Bungabong and coworkers [134]. Furthermore, a study elucidating the physicochemical phenomena underlying the mass transport of DMMP across the functionalized surface-supported LC film in LC-based sensors has been presented [135].

Using a LC-based sensor, Chen and Yang reported a sensor for detecting organophosphates, which are common ingredients in pesticides and chemical warfare agents [136]. They based the sensing on small pH changes during enzymatic hydrolysis of organophosphates using paraoxonase 1 enzyme, which was immobilized on a copper grid that also contains pH-sensitive 5CB doped with 4′-pentyl-biphenyl-4-carboxylic acid (PBA). Disruption of the LC molecules as a result of the enzymatic reaction causes a dark to bright transition that signals the presence of organophosphates. A limit of detection of 1 µM was found using the naked eye. In a related experiment, Zhou et al. reported a new sensor for detecting organophosphate pesticides [76]. Alkaline phosphatase (ALP) was used for the hydrolysis of dichlorvos (DDVP), an organophosphate pesticide. Using ALP-cleavable surfactant sodium monododecyl phosphate (SMP), an orientational transition was observed in the LC molecules in the presence of DDVP and a dark to bright transition was visible. Wang and Yu also reported a pesticide sensor using acetylcholinesterase (AChE) enzyme and Myr [75]. In the absence of the pesticide, a planar orientation was assumed by the LC molecules in the droplet, which gives a bright image. However, there is a bright to dark transition in the presence of AchE-inhibiting pesticides.

**Scheme 3:**
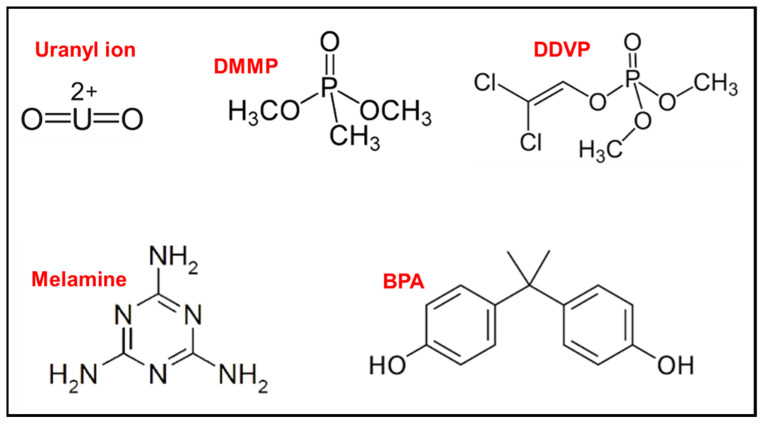
Chemical structures of selected analytes in this section.

Chuang and Chen also reported a LC-based sensor for melamine [137]. Their sandwich sensor system works on the mechanism that melamine, if present, will bind with anti-melamine adsorbed on the glass substrate, which disturbs the LC orientation. Ren et al. has also reported a LC-based aptasensor for the detection of bisphenol A (BPA), an endocrine-disrupting chemical [26]. The BPA analyte forms a complex with the aptamer and this complex disrupts the orientation of the LC molecules from homeotropic to planar, i.e., dark to bright POM image transition. Such a transition is not evident when BPA is not present. They recorded a detection limit of 0.6 fM for BPA with this sensor. A summary of the sensors discussed in this section is presented in Table 3 while Scheme 3 presents the chemical structures of selected analytes in this section.

## 5. Other LC-Based Sensing Applications

Given the simple mechanism on which LC-based sensing is based, such sensors are potentially useful in almost all fields where sensing and continuous analyte monitoring may be required. Therefore, several reports have also appeared in the literature where LCs have been incorporated or used in sensing platforms for a variety of applications. For instance, Hieftje’s research group reported a LC-based fibre-optic chemical sensor for the determination of geometric isomers [138]. The mechanism of determination is based on molecular-geometric-selective absorption of polyaromatic hydrocarbons (PAHs) by the LC. Selective PAH-LC interaction quenches the fluorescence of the LC and such quenching is linearly dependent on the concentration of PAH. The sensor is reversible and reusable and it recorded a detection limit of about 100 nM with a 2 min response time. Dai et al. reported a nitro-substituted pyrrolopyrrole derivative (TPPP-NO_2_) a LC with red-emissive colour [84]. Based on preliminary studies of its characteristic phase transitions, they used the LC as a stimuli-responsive temperature alarm due to its characteristic switching of emission colour from red to yellow at 150–200 °C. Furthermore, LC has also been used to measure ligand-receptor binding events in avidin-biotin interaction [139]. The 5CB molecules used in the sensor were parallel with respect to the plane of the avidin surface, while the orientation changed to randomly oriented nematic domains upon avidin-biotin binding, producing a unique optical response. This kind of sensor should be suitable for studying other ligand–receptor interactions. Szilavi et al. have also shown that the chemical reactivity of bimetallic surfaces like PdAu (on which LC molecules are adsorbed) towards chlorine gas can be tuned by changing the surface composition. According to these researchers, changing the Pd/Au ratio, the dynamic response of the 5CB liquid crystal to chlorine gas adsorption was accelerated three times. In their own experiments, Mistry and coworkers recently showed that LC elastomers can be used as photoelastic strain sensors due to their large stress- and strain-optic coefficients [82]. Uniaxial strains were applied to the elastomers, inducing nematic ordering that was quantified using dye-absorption spectra and polarized Raman spectroscopy. They concluded that the high strain-optic coefficients and high compliances of the elastomers make them suitable candidates for photoelastic coatings for assessing deformations in soft and biomaterials. Similarly, Coskun et al. developed an ultrasensitive strain sensor through patterning of GO liquid crystals on a flexible substrate [140]. They investigated resistive changes in the reduced GO films as a function of uniaxial strain cycles from 0.025–2%. This sensor represents a low cost and sensitive platform for accurately detecting small strains with a detection limit of 0.025%. A liquid crystalline blue phase film has also been used as a photonic shape memory device [141]. The films have narrow photonic band gaps and high reflectivity in the visible region of the electromagnetic spectrum. They obtained multiple blue shift colours via shape memory programming under different mechanical pressures, and quantitative relationships were established between shape change and optical response.

**Figure 8:**
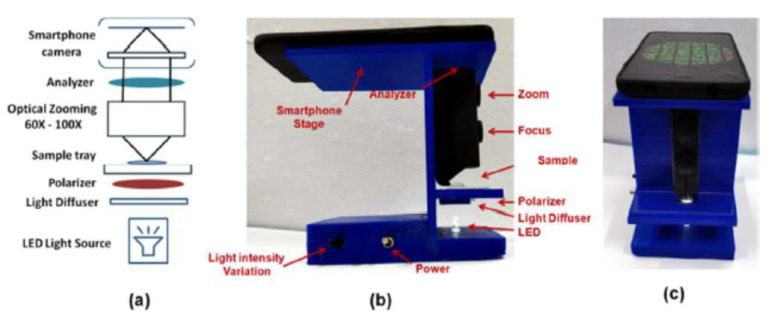
Illustration and photographs of the smartphone-based sensor. (**a**) Schematic diagram of the sensor; (**b**) side view showing the different components of the sensor; (**c**) front view of the sensor. Reprinted with permission from ref. [143]. 2018 The Royal Society of Chemistry.

In a somewhat radical extension of what is possible with LC-based sensing, Cao and coworkers presented a machine learning (ML) framework for optimizing the specificity and speed of chemical sensors that are based on LCs [142]. They showed that ML algorithms can in fact unearth valuable information from orientational transitions induced by different gas-phase analytes and used such data to train accurate and automatic classifiers. Using the ML algorithm to classify thousands of optical images collected from LC responses, they showed that over 99% accuracy is possible with this ML approach compared to about 66% accuracy achievable by using the average brightness of optical images. Furthermore, Nandi and Pal developed a low cost, portable LC-based sensor using a smartphone as a detector [143]. The sensor is a 3D-printed device that used a smartphone for optical detection and its accuracy is similar to those based on POM images (Figure 8). They obtained detection limits of 5 and 10 nM, respectively, for Hb and BSA, and these results are similar to previous data reported from POM images.

**Figure 9:**
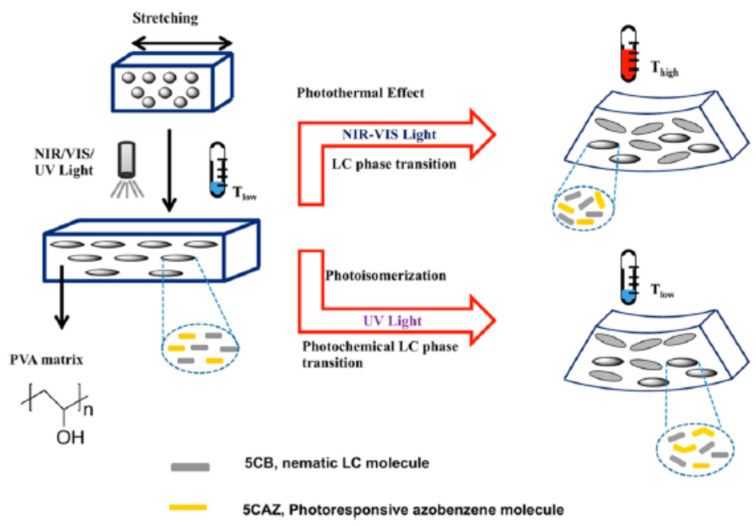
Illustration of the NIR-Vis-UV light-responsive actuator. Reprinted with permission from ref. [144]. 2015 American Chemical Society.

Cheng et al. has developed a new polymer-dispersed liquid crystal (PDLC)/GO nanocomposite films that are light-responsive [144]. They used a combination of photochemical and photothermal processes in the nanocomposite to drive phase transitions in the LCs from nematic to isotropic. Therefore, upon mechanically stretching the film, shape changes and mesogenic alignment took place, making the film respond to NIR-vis-UV light. The GO component acts as a photoabsorbent and nanoscale heat source for converting NIR or visible light into thermal energy. One azobenzene dye incorporated in the LC domains confers UV-responsive property on the nanocomposite films by using photochemical phase transition of the LC upon UV-irradiation. These materials are suitable as actuators and optomechanical devices that are driven by sunlight (see Figure 9). Additionally, deserving mention is that Kimura and coworkers used thermotropic LCs as membrane materials and neutral carriers to investigate the effect of an ordered arrangement of neutral carriers on the property of the resulting ion sensors [145], while Votava and Ravoo presented a tutorial review on cyclodextrin-based LCs as versatile supramolecular materials [146].

## 6. Future Perspectives on LC-Based Sensors

Almost all the LC-based sensors discussed in the preceding sections are based on 5CB liquid crystal; only a few sensors used different LCs. Perhaps this is due to the fact that 5CB is the most extensively characterized and written-about in the literature and it is readily available, however, it will be interesting to see another one or two members of the alkylcyanobiphenyl LCs being used in LC-based sensors. For instance, 6CB, the closest member to 5CB will be seen in publications in the near future. 7CB and heavier members may be intractable in LC-based sensors because unlike 5CB and 6CB, these heavier members do not exist as liquids at room temperature. Therefore, it will be interesting to see how 6CB performs in LC-based sensing in the near future. While some of the sensors discussed in this review used a mixture of LCs, probably to achieve the expected optical response from the LC, the majority of the works reviewed here used only one LC, which is 5CB. What happens if a mixture of two or three LCs are used? Perhaps a mixture of 5CB and 6CB can be given a trial in a LC-based sensor and it may give an even better optical readout. It is not clear what specific properties the mixture will display, one expects that the phase transitions may be different from either of the components with an attendant modification to the birefringence properties of the mixture, which may enhance the optical readout when such a mixture is used in a sensor.

Given the simplicity and low-cost nature of LC-based sensors, it is not impossible to expect more of such sensors use smartphone detectors in the future. As stated in ref. [143], the results from a smartphone-based sensor are comparable to those from POM images. This shows what is possible with LC-based sensing: the incorporation of smartphones to make the detection easier even for the untrained person. Therefore, the stage is set for the incorporation of smartphones into LC-based sensing. As seen with the results presented in ref. [142], machine-learning has the capability to further push the envelope in LC-based sensing. The impact of ML can be revolutionary, not only with LC-based sensors but across the entire field of experimental science. With a specific focus on LC-based sensors, the detection accuracy obtainable with an ML framework is unrivalled currently by data obtained from POM images and average brightness. Furthermore, as stated in ref. [142], high accuracies were also achieved using time snapshots that were collected early in the LC response, thus opening an avenue for fast sensors. The implication of this is that by incorporating ML into LC-based sensing more accurate data can be generated at much faster rates. Therefore, this author foresees the involvement of more and more ML and artificial intelligence (AI) incorporated into scientific research, data generation and analysis.

## 7. Conclusions

This review has presented a comprehensive analysis of the development and applications of LC-based sensors. It was shown that the heart of every LC-based sensor is the liquid crystal material, which is 5CB in most cases. It is the orientational transitions that the LC molecules undergo that present the optical signal that is measured. The trigger of the orientational transition is some form of interaction between the analyte of interest and a component of the sensor. Such interactions may trigger a realignment of the LC molecules directly or it may produce a new product that then triggers orientational transition of the LC molecules. The repertoire of applications of these sensors is distinct on the basis of the component of the sensor that is responsible for interacting with the analyte. In some cases, it could be a modification of the surfactant or the substrate surface that will engender some form of analyte interaction, while in others an independent molecule such as an aptamer that is specific for the analyte of interest is incorporated into the sensor. Common application areas of LC-based sensors are in biosensing such as glucose monitoring, biomarker detection, detection of metabolites, protein and peptide detection, antigen-antibody interaction monitoring, nucleic acid detection and so on. Furthermore, LC-based sensors have been developed for heavy metal detection, pH monitoring and detection of VOCs. In addition, these sensors have been applied to gas sensing, detection of toxic agents as well as stress detection and other applications in material science. A smartphone was used for detection in one publication and it is expected that such a detection approach in LC-based sensing will increase. The incorporation of ML and AI into LC-based sensing were also briefly discussed in this review and it is expected these will play increasing roles in this type of sensing platform.

## Figures and Tables

**Table 1:** List of LC-based biosensors *.

LC Material	Sensing Platform	Analyte	Mode of Interaction with Sensor	Detection Method	Ref.
5CB	UV-treated 5CB placed inside a GOx-modified gold grid	Glucose	The H^+^ produced from the oxidation of glucose triggered an optical response of the LCs from dark to bright	POM	[48]
5CB	OTAB-treated 5CB in copper grid	Glucose and H_2_O_2_	ssDNA adsorbed onto nano CeO_2_ gets dislodged from the surface in the presence of H_2_O_2_ or glucose	POM	[49]
5CB	5CB functionalized with PAA-b-LCP and QP4VP-b-LCP	Glucose	Presence of glucose triggers reorientation of the LC at the pH-sensitive LC-aqueous interface	POM	[46]
5CB	APBA-decorated 5CB microdroplets	Glucose	Binding between glucose and APBA on 5CB induced reorientation of the LC molecules	POM	[37]
Polymer dispersed LC composites	Micrometric droplets of PDLC composites prepared by encapsulation of L-ChAc PVAB	Glucose, amino acid and DNA	Interaction of glucose with droplets leaves behind a “chicken-skin texture with rare light spots”	POM	[50]
5CB	5CB sandwiched between 2 glass slides treated with cTnI antibody and DMOAP/APTES	Cardiac troponin I (cTnI)	Target-cTnI antibody interaction triggers LC reorientation	POM	[51]
5CB	5CB decorated with a nonionic surfactant dodecyl β-D-glucopyranoside	BSA, Con A and lysozyme	Interaction of analyte with the LC/aqueous interface causes LC reorientation	POM	[90]
5CB	5CB in TEM grid cell on treated glass	BSA, ChTg Hb and lysozyme	Interaction of protein with LC/aqueous interface triggers LC rearrangement	POM	[91]
5CB	5CB in PDMAEMA-treated TEM grid cell	BSA	Electrostatic interaction between BSA and PDMAEMA triggers LC reorientaton	POM	[92]
5CB	5CB droplets functionalized with PAA-b-LCP	Avidin	Avidin–biotin binding at the 5CB/aqueous interface causes LC rearrangement	POM	[93]
5CB	5CB coated with biotinylated lipids and biotinylated anti-goat IgG in TEM grid cell	Goat IgG	Interaction of goat IgG with the functionalized LC molecules causes LC reorientation	POM	[65]
5CB	5CB sandwiched between DMOAP and APTES/ DMOAP-treated glass slides	carcinoembryonic antigen (CEA)	Reorientation of the LC molecules due to specific interaction between CEA and modified glass slide	POM	[66]
5CB	5CB decorated with quaternary ammonium-based gemini surfactants	BSA, lysozyme and trypsin	Interaction of goat IgG with the functionalized LC molecules causes LC reorientation	POM	[94]
E7	Surfactin-decorated LC on DMOAP-treated glass slide	Secondary structure of Cyto, BSA, Hb, Con A and fibronectin	Reorientation of LC due to interaction between protein and LC/aqueous interface	POM	[95]
DLC	DLC sandwiched between two treated glass slides	BSA	Absorption of azobenzene chromophore for concentration determination and interaction between BSA and LC/aqueous interface that causes LC reorientation	Absorption and POM	[52]
5CB	5CB sandwiched between two glass slides	Anti-biotin IgG	LC reorientation caused by specific binding between a target anti-biotin IgG and biotinylated BSA	POM	[56]
5CB	5CB sandwiched between DMOAP/APTES-treated glass slides	HBD-2	LC reorientation due to specific binding of anti-HBD-2 antibody and HBD-2	POM	[55]
5CB	5CB mixed with SDS in copper grid placed on DMOAP-coated glass slide	P53 gene	Interaction of p53 with DNA dendrimers induced rearrangement of LC molecules	POM	[96]
5CB	5CB placed between two treated glass slides functionalized by droplets of DNA or PNA	DNA	Reorientation of LC molecules due to DNA interacting with the PNA	POM	[63]
5CB	5CB-filled copper grids immersed in OTAB	DNA SSBs	Reorientation of LC due to decreased electrostatic interaction between SSBs and cationic surfactant, OTAB	POM	[64]
5CB	Aptamer on DMOAP/APTES-treated glass slide consisting of 5CB	Interferon-γ (IFN-γ)	Reorientation of LC as a result of aptamer-IFN-γ binding	POM	[97]
5CB	Tuberculous antigens immobilized on treated surfaces in the presence of 5CB	Tuberculosis antibody	Reorientation of LC due to antigen-antibody interaction	POM	[98]
5CB	5CB immobilized with complementary probe DNA	PSA, CEA and AFP	Reorientation of the LC when target DNA hybridizes with the complementary probe DNA	POM	[67]
5CB	5CB droplets functionalized with Herceptin antibody	SK-BR3 cancer cells	Selective interaction of the LC with SK-BR3 induces orientational change	POM	[99]
5CB	PAA-b-LCP functionalized with urease in the presence of 5CB in TEM grid on an OTS-coated glass	Urea	LC orientational change caused by pH change due to urea hydrolysis	POM	[58]
5CB	Droplets containing 5CB with urease-functionalized PAA-b-LCP	Urea	Reorientation of the LC due to pH change caused urea hydrolysis	POM	[57]
5CB	Stearic acid-doped 5CB microdroplets in the presence of urease	Urea	Reorientation of LC due to deprotonation of stearic acid as a result of urea hydrolysis	POM	[35]
5CB	UV-treated 5CB placed in copper grids on OTS-treated glass	Urease	Ammonia produced from urea hydrolysis in the presence of urease induces orientational change	POM	[61]
5CB	5CB- filled Copper TEM grid placed on functionalized glass	Urease and Cu(II)	LC reorientation due to urea hydrolysis or urease inhibition in the presence of Cu(II)	POM	[100]
5CB	Droplets of 5CB doped with stearic acid on microscope slides	Urease	Reorientation of LC due to deprotonation of stearic acid as a result of urea hydrolysis	POM	[101]
5CB	DOPG-decorated LC with poly-L-lysine	Trypsin	Reorientation due to interaction of trypsin with the LC/aqueous interface	POM	[102]
5CB	5CB-DTAB in the presence of BSA	Trypsin	Reorientation of the LC on adding BSA, and LC alignment persists in the presence of trypsin	POM	[54]
5CB	BSA-modified grid was filled with 5CB	Trypsin	Reorientation of 5CB at the LC/aqueous interface due to BSA hydrolysis	POM	[53]
5CB	A cationic surfactant-decorated 5CB on OTS-treated glass	AChE and its inhibitors	Reorientation of LC at the LC/aqueous interface due to AChE	POM and time-dependent Br	[60]
5CB	5CB doped with glyceryl trioleate in gold grid cell on OTS-treated glass	Lipase	LC reorientation due to product trioleic acid interacting with LC/aqueous interface	POM	[103]
5CB	5CB and a monolayer of phospholipids in TEM grid on treated glass	Lipase	LC realignment due to hydrolysis of phospholipids	POM	[103]
5CB	OTB monolayer on 5CB inside copper grid cells placed on OTS-treated glass	Carboxylesterase (CES)	Disruption of LC orientation due to hydrolysis of OTB	POM	[62]
5CB	PBA-doped 5CB microdroplets	Penicillinase	LC reorientation due to deprotonation of PBA at the aqueous/LC interface	POM	[104]
5CB	5CB doped with C12-aldehyde in copper grids placed on OTS-treated glass	Catalase	LC reorientation due to interactions between hydrogen peroxide and 5CB doped with C12-aldehyde	POM	[105]
5CB	5CB functionalized with dodecyl β-_D_-glucopyranoside	Cellulase and cysteine	Reorientation of LC due to enzymatic hydrolysis between cellulase and the surfactant	POM	[106]
5CB	5CB doped with DOPG in the presence of PLA in grid cells	Thrombin	Hydrolysis of PLA by thrombin causes a disruption of the LC/aqueous interface	POM	[107,108]
5CB	5CB-filled TEM grids or 5CB droplets in the presence of surfactant	Cholic acid	LC realignment due to competitive adsorption of cholic acid at the LC/aqueous interface	POM	[109,110]
5CB	5CB droplets in the presence of surfactant	Bile acids	Orientational transition due to competitive interaction of bile acids at the LC/aqueous interface	POM	[111,112]
5CB	PAA-b-LCP-coated 5CB in the presence of ChOx and HRP	Cholesterol	Oxidation of cholesterol disrupts the LC/aqueous interface		[113]
5CB	UV-treated 5CB placed in grid cells on treated glass	Cholesterol	H^+^ generated by reaction of ChOx with cholesterol disrupts the LC alignment		[114]
5CB	5CB on polymeric surface in the presence of antibodies	Viruses	Antibody-virus binding induces reorientation of LC molecules		[70]
5CB	5CB covered with LPS monolayers in gold grid cells	Bacteria	Reorientation of LC due to interaction of bacteria with the LC/aqueous interface		[116]
5CB	5CB in TEM grid cells layered with LPS	PG and LTA	Disruption of LC alignment due to PG/LTA interacting with the LC/aqueous interface		[117]
5CB	Phospholipid monolayer on 5CB contained in copper grid in the presence of CS-GO	*E. coli*	CS-GO action on bacteria may or may not disrupt the LC/aqueous interface, depending on bacterial viability		[118]
5CB	5CB on cell-covered glass slide with PDL-coated glass top	Neurons, fat cells and muscle cells	Preferential orientation of LC molecules on cells provided well-resolved images	Phase contrast images	[69]
LI-1565	LC filled in sample cells	Plant pathogens	Perturbation of LC alignment in the presence of plant pathogen	POM and dielectric measurements	[119]
5CB	5CB-filled TEM grid cell functionalized with DTAB in the presence of DNA	Myricetin	DNA degradation by myricetin causes reorientation of the LC molecules	POM	[120]
5CB	5CB sandwiched between two glass slides in the presence of RAC aptamers	Ractopamine (RAC)	Formation of AuNP-RAC-aptamer conjugate disrupts the LC alignment	POM	[121]
5CB	BSA-aflatoxin on DMOAP/APTES-treated glass slide in the presence of 5CB and AFT-antibody	Aflatoxin (AFT)	Competitive binding between AFT and BSA-AFT for the antibody disrupts the LC alignment	POM	[122]
5CB	5CB sandwiched between two glass slides in the presence of aptamer	Tetracycline	Aptamer-tetracycline interaction disrupts LC alignment	POM	[123]

* GOx: glucose oxidase; POM: polarized optical microscopy; OTAB: octadecyltrimethyl ammonium bromide; ssDNA: single-stranded DNA; PAA; poly(acrylicacid-b-4- cynobiphenyl-4′-oxyundecylacrylate); LCP: liquid crystal polymer; QP4VP: quaternizedpoly(4-vinylpyridine-b-4-cynobi-phenyl-4′-oxyundecylacrylate; APBA: 3-Aminophenyl boronic acid; PDLC: polymer dispersed liquid crystal; L-ChAc: cholesteryl acetate; PVAB: polyvinyl alcohol boric acid; DMOAP; *N*,*N*-Dimethyl-*N*-octadecyl-3-aminopropyltrimethylsilane ammonium chloride; APTES: (3-aminopropyl)triethoxysilane; BSA: bovine serum albumin; Con A: concanavalin A; ChTg: α chymotrypsinogen-A; Hg; haemoglobin; PDMAEMA: Poly(2-(dimethylamino)ethyl methacrylate); IgG: Imunoglobulin G; E7: a mixture of cyano-biphenyl and terphenyls; HBD-2: β-defensin-2; DNA SSBs: DNA single-strand breaks; PSA: prostate specific antigen; CEA: carcinoembryonic antigen; AFP: alpha-fetoprotein; OTS: octadecyltrichlorosilane; DOPG: (dioleoyl-sn-glycero-3-phospho-rac-(1-glycerol) sodium salt); DTAB: dodecyl trimethyl ammonium bromide; AChE: acetylcholinesterase; BR: bright area coverage ratio; ChOx: cholesterol oxidase; HRP: horseradish peroxidase; LPS: lipopolysaccharides; PG: peptidoglycan; LTA: lipoteichoic acid; CS–GO: chitosan–graphene oxide; AuNP: gold nanoparticles.

**Table 2:** List of LC-based sensors of heavy metal ions, nitrite and pH *.

LC Material	Sensing Platform	Analyte	Mode of Interaction with Sensor	Detection Method	Ref.
5CB	UV-treated 5CB placed inside a urease-modified gold grid	Cu(II)	OH^−^ ions from ammonia product of urea hydrolysis deprotonated the UV-treated LC thereby causing an orientational transition of the LC; the presence of Cu(II) inhibits this transition	POM	[124]
5CB	Stearic acid-doped 5CB on an OTS-coated glass	Ca(II), Mg(II), Cu(II) and Co(II)	Interaction between metal ion and deprotonated stearic-acid molecules causes an orientational transition of 5CB at the LC/aqueous interface	POM	[72]
5CB	Pb(II)-specific DNAzyme incorporated on DMOAP/APTES treated glass in the presence of 5CB doped with AIE luminogen	Pb(II)	Fluorescence intensity change due to LC reorientation at the LC/aqueous interface as induced by DNAzyme and its catalytic cleavage in the presence of Pb(II)	Fluorescence	[71]
5CB	5CB in TEM grid in the presence of CTAB, SRNA and aptamer	Pb(II)	Formation of a more stable quadruplex structure of the RNA with Pb(II) thereby causing a reorientation of the LC at the LC/aqueous interface	POM	[73]
5CB	5CB incubated with magnetic nanoparticles were dispensed in a gold grid placed on a treated glass substrate	Pb(II)	Reorientation of LC at the LC/aqueous interface caused by interaction of Pb(II) with abundant hydroxyl groups on the surface of the nanoparticles	POM	[125]
5CB	5CB droplets consisting of OTAB pre-incubated with an aptamer specific for Hg(II)	Hg(II)	Orientational transition of the LC in the presence of Hg(II) due to weakening of OTAB-aptamer electrostatic interactions by the Hg(II) ions	POM	[126]
5CB	MeDTC-doped 5CB in TEM grids on OTS-treated glass slide	Hg(II)	Reorientation of the LC molecules due to complexation between the chelating group of MeDTC and Hg(II) ions	POM	[27]
5CB	5CB functionalized with PAA-b-LCP in Cu grids	Ca(II)	Reorientation of LC molecules due to complexation of PAA chains of PAA-b-LCP with the metal ions	POM	[127]
5CB	Decylaniline-doped 5CB placed in TEM grid on a glass substrate	Nitrite ion	Reorientation of 5CB due to reaction between nitrite and decylaniline to yield diazonium ions	POM, image analysis	[128]
5CB	5CB doped with a pH-sensitive molecule in Cu grid placed on a glass substrate	H^+^	Realignment of LC molecules at the LC/aqueous interface due to dissociation of the dopants	POM	[80]
MLC-2132 doped with CB15	CLC DEDs coated with pH-responsive PAA-b-LCP	H^+^	Reorientation of LC due to deprotonation and protonation of the carboxylate on the PAA chain	POM	[81]

* POM: polarized optical microscopy; OTS: octadecyltrichlorosilane; DMOAP; *N*,*N*-Dimethyl-*N*-octadecyl-3-aminopropyltrimethylsilane ammonium chloride; APTES: (3-aminopropyl)triethoxysilane; AIE: aggregation-induced emission; CTAB: cetyltrimethylammonium bromide; SRNA: spinach RNA; OTAB: octadecyltrimethyl ammonium bromide; MeDTC: *N*-dodecyldithiocarbamate; PAA-b-LCP: poly(acrylic acid)-b-poly(4- cyanobiphenyl-4′-oxyundecylacrylate); CLC: cholesteric LC; CEDs: double emulsion droplet; CB15: (S)-4-cyano-4 -(2-methylbutyl)biphenyl.

**Table 3:** List of LC-based sensors for gases, VOCs and toxic substances *.

LC Material	Sensing Platform	Analyte	Mode of Interaction with Sensor	Detection Method	Ref.
E7	E7 droplets deposited onto the array of gold-coated micro-pillars	NO_2_	Transport of NO_2_ molecules through the LC film to the LC–gold interface induces an orientation transition in the LC film	Transmitted light or image brightness	[77]
Cholesteric LC mixture EE1	LC doped with magnetite nanoparticles intercalated into alumina matrix	CO	Shift in the selective reflection peak wavelength due to interaction between CO molecules and magnetite nanoparticles dispersed in the liquid crystal	Shift in transmission peak	[79]
5CB	5CB placed in grid cells on chitosan-Cu(II)-decorated glass substrate		Reorientation of the LC molecules due to competitive binding between ammonia gas and Cu(II) on the glass substrate	POM	[129]
5CB	LC/polymer composite fibres spread out as a mat on a glass substrate	Acetone and toluene	Change in the optical properties of the LC/fibre mats upon absorption of analyte gases	Transmittance	[131]
N * LC	N * LC encapsulated in microscale PVP fibers	CO_2_	Change in pitch length of sensor due to selective chemical reaction between dopant and analyte	Reflectivity pitch length measurements	[78]
CLC	CLC film coated side polished fiber (CLCFC-SPF)	Acetone, methanol and THF	Resonant dips in the transmitted spectrum as VOC gases interact with the CLCFC-SPF sensor	Shifts in transmittance peaks	[130]
5CB	LA-5CB in TEM grid placed on a glass slide	Butylamine vapour	Orientational transition of LC triggered by a reaction between LA and butylamine	POM	[133]
5CB	UO_2_^2+^–dependent DNAzyme, its substrate, a capture probe and 5CB sandwiched between DMOAP- and TEA-treated glass slides	UO_2_^2+^ (Uranyl ion)	Reorientation of LC when the cleaved product released from DNAzyme hybridizes with capture probe to form a duplex	POM	[74]
5CB	5CB films consisting of Cu(II) ions applied to functionalized substrates	DMMP vapour	Reorientation of LC molecules due to capture of DMMP by Cu(II) ions	POM	[15,134]
5CB	5CB films consisting of Al(III) ions applied to functionalized substrates	DMMP vapour	Reorientation of LC molecules due to interaction between DMMP and aluminium perchlorate-decorated surface	POM	[135]
5CB	5CB doped with PBA in the presence of PON1 on Cu grid	Organophosphates	Reorientation of LC molecules due to pH changes caused by enzymatic hydrolysis of organophosphates	POM	[136]
5CB	5CB droplets doped with ALP and SMP	Organophosphate pesticide, DDVP	Reorientation of LC molecules due to DDVP hydrolysis by ALP	POM	[76]
5CB	5CB droplets doped with AChE and Myr	Baycarb and dimethoate (pesticides)	Reorientation of 5CB at the LC/aqueous interface due to inhibition of Myr hydrolysis in the presence of pesticide	POM	[75]
5CB	5CB sandwiched between DMOAP-treated glass slides to one of which biotin-labelled anti-melamine is immobilized	Melamine	Reorientation of LC molecules due to biding of melamine and anti-melamine linked with streptavidin to the primary anti-melamine on the substrate	POM	[137]
5CB	5CB sandwiched between APTES/DMOAP-treated glass slides in the presence of BPA aptamer	BPA	Reorientation of LC molecules due to formation of aptamer-BPA complex	POM	[26]

* E7: a mixture of cyano-biphenyl and terphenyls; EE1: consists of multiple chiral cyano-biphenyl, cyano-terphenyl derivatives; POM: polarized optical microscopy; N * LC: chiral-nematic liquid crystals; PVP: polyvinylpyrrolidone; CLC: cholesteric liquid crystal; CLCFC-SPF: cholesteric liquid crystal film coated side polished fibre; LA: lauric aldehyde; DMOAP: *N*,*N*-Dimethyl-*N*-octadecyl-3-aminopropyltrimethylsilane ammonium chloride; TEA: triethoxybutyrylsilane; DMMP: dimethyl methylphosphonate; PBA: 4-pentyl-biphenyl-4′-carboxylic acid; PON1: paraoxonase 1; DDVP: dichlorvos; ALP: alkaline phosphatase; SMP: sodium monododecyl phosphate; AChE: acetylcholinesterase; Myr: myristoylcholine; BPA: bisphenol A; APTES: (3-Aminopropyl)triethoxysilane.

## Data Availability

Not applicable.
